# The real-world safety of Nivolumab: a pharmacovigilance analysis based on the FDA adverse event reporting system

**DOI:** 10.3389/fimmu.2025.1605958

**Published:** 2025-05-26

**Authors:** Yutong Wu, Yue Zhou, Shiyue Xia, Zhaoyou Meng

**Affiliations:** ^1^ Department of Neurology, Second Affiliated Hospital of Army Medical University, Chong Qing, China; ^2^ Basic Medical College, Army Medical University, Chong Qing, China

**Keywords:** nivolumab, FAERS, mm, NSCLC, pharmacovigilance, adverse events

## Abstract

**Background:**

Nivolumab, a human immunoglobulin IgG4 monoclonal antibody targeting PD-1 receptor, received initial FDA approval in 2014 for treating unresectable or metastatic malignant melanoma (MM), followed by approval for metastatic squamous and non-squamous non-small cell lung cancer (NSCLC) in 2015. With expanding clinical applications of nivolumab, comprehensive evaluation of its safety profile in real-world healthcare settings becomes increasingly crucial.

**Methods:**

We compiled a real-world safety dataset of nivolumab from the FDA Adverse Event Reporting System (FAERS) database, encompassing reports from Q4–2014 through Q2 2024. To evaluate the association between nivolumab and adverse events (AEs), we employed four distinct disproportionality analysis methods: Reporting Odds Ratio (ROR), Proportional Reporting Ratio (PRR), Multi-item Gamma Poisson Shrinker (MGPS) and Bayesian Confidence Propagation Neural Network (BCPNN). Additionally, we utilized Weibull distribution modeling to characterize the temporal risk patterns of identified adverse events.

**Results:**

Our analysis identified 64,627 AEs reports associated with nivolumab. The most frequently reported AEs included fatigue, dyspnea, musculoskeletal pain, decreased appetite, cough, nausea, and constipation. Notably, we detected several potential safety signals not currently listed in the prescribing information: Malignant neoplasm progression, weight decreased, sepsis myocarditis, encephalitis and hypotension.

**Conclusions:**

Our large-scale pharmacovigilance study identified significant safety signals associated with nivolumab, including previously unrecognized adverse drug reactions. The identification of these safety signals underscores the importance of ongoing post-marketing surveillance for immune checkpoint inhibitors. Future studies should investigate the mechanisms underlying these associations and develop targeted monitoring protocols.

## 1 Introduction

Nivolumab, a human monoclonal antibody targeting programmed cell death protein 1 (PD-1), represents a breakthrough in cancer immunotherapy with its unique immunomodulatory properties. As an immune checkpoint inhibitor, it has been approved for treating various advanced or metastatic malignancies (1). The PD-1 pathway plays a crucial role in immune regulation. PD-1, expressed on activated T cells, serves as a key checkpoint molecule that downregulates T-cell responses through multiple mechanisms. Many tumors exploit this pathway by upregulating PD-1 ligands (PD-L1/PD-L2), creating an immunosuppressive microenvironment that evades T-cell-mediated immune surveillance. Nivolumab specifically binding to PD-1 receptors, thereby blocking their interaction with both PD-L1 and PD-L2. This inhibition releases the PD-1 pathway-mediated suppression of immune responses and exerts its antitumor therapeutic effects (2).

Nivolumab received FDA approval in 2014 for treating unresectable or metastatic advanced melanoma (MM). MM represents the most lethal form of skin cancer, originating from malignant transformation of melanocytes (3). These neural crest-derived cells are normally distributed in the leptomeninges, uvea, brain parenchyma, mucous membranes, and skin (4). The anatomical distribution of melanocytes correlates with potential melanoma development sites. For primary CNS malignant melanoma(MM), three pathogenic origins have been proposed: (1) mesoderm-derived pigment cells migrating via leptomeningeal vasculature; (2) abnormal embryonic ectodermal cell origins; (3) neural crest-derived melanocytic precursors (5). While gross total resection or subtotal resection significantly improves survival, combination non-surgical therapies remain the optimal approach for unresectable cases. Monotherapies (ipilimumab, nivolumab, pembrolizumab) and combination ipilimumab-nivolumab have demonstrated clinical efficacy in advanced MM, improving 5-year survival rates from <5% to approximately 30% in patients receiving immunotherapy or targeted therapies (6–8). The phase III CheckMate 067 trial established nivolumab plus ipilimumab as achieving the longest median overall survival (72.1 months) among phase III melanoma studies to date (9). Furthermore, this trial demonstrated superior 5-year overall survival rates for combination therapy (52%) and nivolumab monotherapy (44%) versus ipilimumab alone (26%) (10).

Nivolumab received FDA approval in 2015 for the treatment of metastatic squamous or non-squamous non-small cell lung cancer (NSCLC). Lung cancer remains the leading cause of global cancer-related mortality, with NSCLC exhibiting particularly dismal cure rates and survival outcomes, especially when presenting as metastatic disease (11). The high mortality stems from frequent metastatic dissemination at diagnosis, underscoring the critical need for more effective systemic therapies to improve long-term survival (12, 13). Current immune checkpoint blockade (ICB) strategies approved or under development for NSCLC include anti-PD-1 antibodies (nivolumab, pembrolizumab) and anti-PD-L1 antibodies (atezolizumab, durvalumab, avelumab) (14). The CheckMate 816 trial demonstrated that neoadjuvant nivolumab plus chemotherapy significantly improved complete response rates (24% vs. 2.2%, P < 0.001) and event-free survival (HR 0.68, 95% CI 0.49-0.93) compared to chemotherapy alone, with enhanced benefits observed in patients exhibiting ≥1% PD-L1 tumor expression (15). Furthermore, perioperative nivolumab-chemotherapy combination therapy yielded higher rates of pathological complete response and prolonged survival compared to chemotherapy alone in resectable Stage IIIA or selected IIIB NSCLC patients (16).

Nivolumab has been widely used in clinical practice, particularly for the treatment of MM and NSCLC, demonstrating significant survival benefits in these patient populations. Given its broad application, understanding the safety profile of nivolumab in real-world settings is crucial. According to the prescribing information, the most frequently reported adverse reactions include fatigue, dyspnea, musculoskeletal pain, decreased appetite, cough, nausea, and constipation. However, clinical trials typically enroll specific patient populations with strict inclusion and exclusion criteria, meaning that the adverse events (AEs) listed in the prescribing information may only reflect those observed in a selected subset of patients. To further evaluate the real-world safety of nivolumab, this study analyzed data from the FDA Adverse Event Reporting System (FAERS) database. This comprehensive analysis provides additional evidence to guide healthcare professionals in the clinical use of nivolumab, complementing findings from controlled clinical trials.

## 2 Materials and methods

### 2.1 Data source and processing

**Figure 1:**
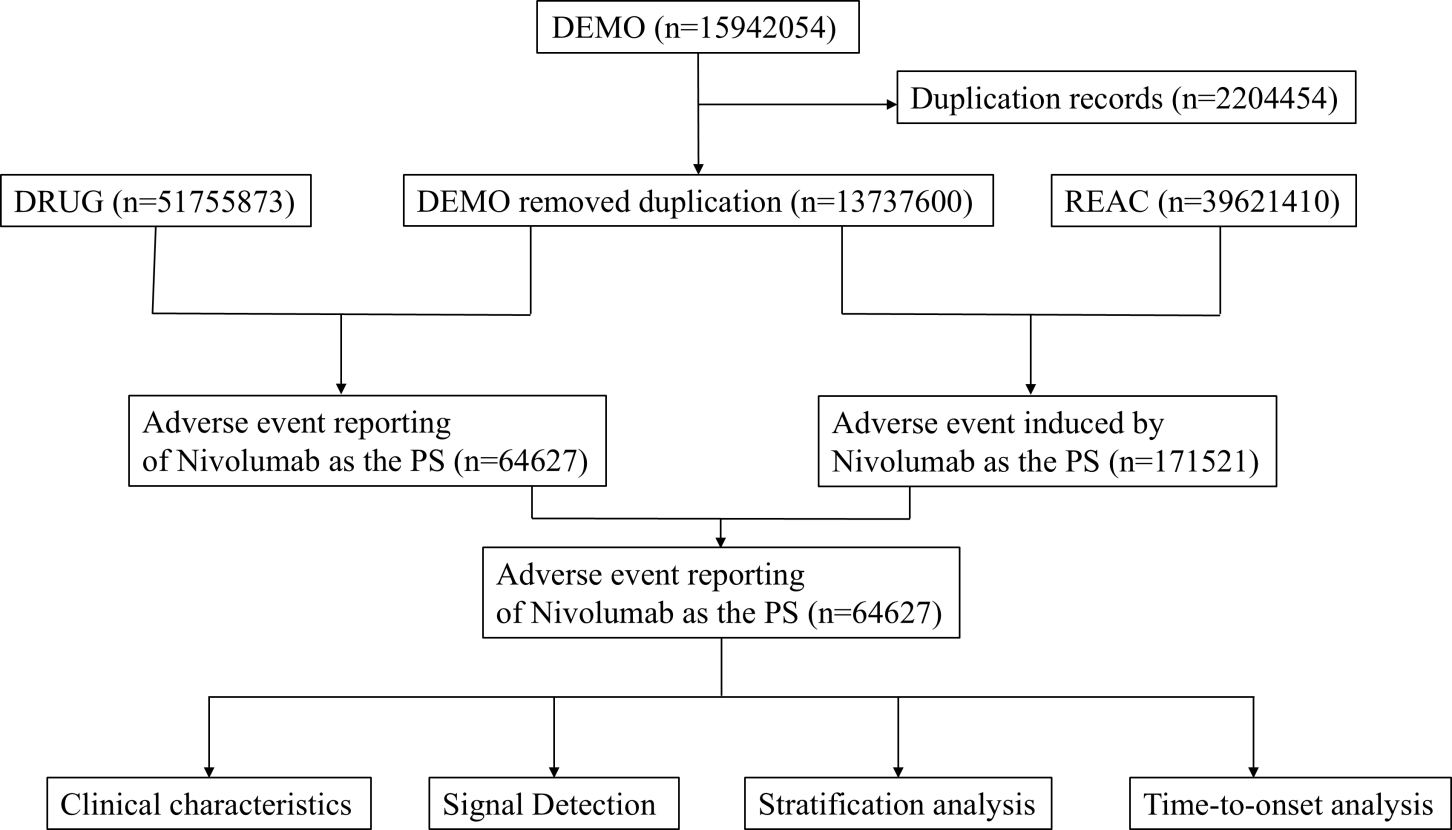
Flow-process diagram of Nivolumab-related AEs from FAERS database.

This study compiled AE reports from the FAERS database, spanning from the fourth quarter of 2014 to the second quarter of 2024. Only reports where nivolumab was designated as the primary suspect (PS) drug were included. Data extraction and cleaning were performed using R software (version 4.4.1). The initial database contained 15,942,054 reports, from which 2,204,454 duplicate entries were excluded in accordance with FDA guidance (17). Key fields, including PRIMARYID, CASEID, and FDA_DT, were extracted from the original dataset and sorted. For reports sharing the same CASEID, only the entry with the most recent FDA_DT was retained. In cases where CASEID and FDA_DT were identical, the record with the highest PRIMARYID was selected. AEs were classified based on Preferred Term (PT) and System Organ Class (SOC) using Medical Dictionary for Regulatory Activities (MedDRA, version 27.0) (18). **Figure 1** illustrates the flowchart of nivolumab-associated AEs identified in the FAERS database.

### 2.2 Data analysis

In this study, we employed multiple pharmacovigilance methodologies including the reporting odds ratio(ROR) (6–8), proportional reporting ratio(PRR) (19), multi-item gamma Poisson shrinker (MGPS) (20), and Bayesian confidence propagation neural network (BCPNN) (21) to evaluate significant associations between nivolumab and adverse events (AEs). AEs exceeding the positivity threshold in at least one of these methods were identified as potential signals. **Supplementary Table S1** presents the detailed 2×2 contingency matrices, while **Supplementary Table S2** provides the specific parameters for the four primary signal detection algorithms. Furthermore, Weibull distribution modeling was applied to analyze the time-to-onset of adverse events.

## 3 Results

### 3.1 Basic characteristics of AEs and population

Our analysis encompassed 64,627 adverse event (AE) reports associated with nivolumab. **Table 1** presents the baseline characteristics of these reports. Among reporters, males constituted a significantly higher proportion (36,457, 56.4%) compared to females (19,698, 30.5%). The elderly population (65–85 years) represented the largest age group reporting AEs (22,802, 35.3%), followed by adults aged 18–65 years (20,038, 31.0%). Consistent with clinical indications, the primary reported use of nivolumab was for NSCLC (9,809 cases, 15.2%), with MM being the secondary indication (8,020 cases, 12.4%). The FAERS database revealed death as the most frequently documented serious outcome (18,616 cases, 28.8%), followed by hospitalization, life-threatening events, and disability. Geographically, the United States contributed the majority of reports (27,714, 42.9%), with Japan ranking second (10,656, 16.5%). Healthcare professionals submitted the largest proportion of reports (20,176, 31.2%), followed by consumers (15,887, 24.6%). Following FDA approval of nivolumab for MM in 2014, AE reports demonstrated an annual increase, peaking in 2019 (9,533 cases). Subsequently, reports declined yearly, reaching 3,626 cases in 2024. But our analysis only included data from the first two quarters of 2024.

**Table 1:** Clinical characteristics of Nivolumab adverse event reports from the FAERS database (Q4 2014 – Q3 2024).

Characteristics	Case numbers	Case proportion (%)
Number of events	64627	
Gender		
Male	36457	56.4
Female	19698	30.5
Miss	8472	13.1
Age		
<18	444	0.7
18-65	20038	31.0
65-85	22802	35.3
>85	735	1.1
Miss	20608	31.9
Top 3 Indication		
Non-small cell lung cancer	9809	15.2
Malignant melanoma	8020	12.4
Renal cell carcinoma	4215	6.5
Outcome		
Death	18616	28.8
Hospitalization	17926	27.7
Life-Threatening	2777	4.3
Disability	354	0.5
Top 5 Reported Countries		
United States	27714	42.9
Japan	10656	16.5
France	5785	9.0
Germany	3132	4.8
China	2020	3.1
Reporter		
Doctor of Medicine	20176	31.2
Consumer	15887	24.6
Healthcare Professional	12089	18.7
Pharmacist	4068	6.3
Reporting year		
2014-2016	7692	11.9
2017	7458	11.5
2018	8071	12.5
2019	9533	14.8
2020	8256	12.8
2021	7944	12.3
2022	7431	11.5
2023	4616	7.1
2024	3626	5.6

### 3.2 Signal detection related to SOC levels

**Figure 2:**
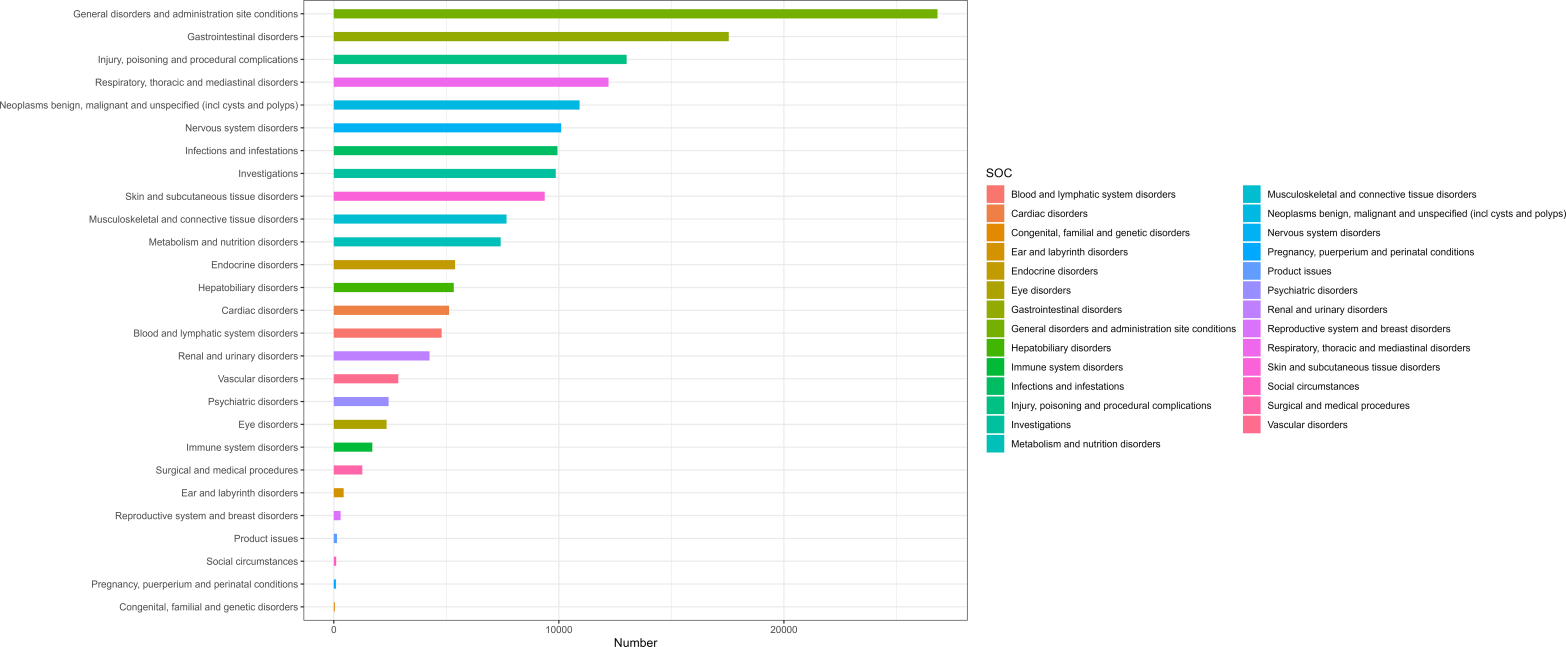
AEs related to the system organ class (SOC) level.

**Table 2** presents nivolumab-associated AEs across all 27 SOCs, while **Figure 2** illustrate the signal strengths at the SOC level in the FAERS database. The most frequently reported category was general disorders and administration site conditions [n=26,822, ROR (95% CI) = 0.85 (0.84-0.86)], whereas the strongest signal intensity was observed for endocrine disorders [n=5,387, ROR (95% CI) = 13.18 (12.82-13.55)]. Several additional SOCs demonstrated robust signal detection, including gastrointestinal disorders [n=17,549, ROR (95% CI) = 1.25 (1.23-1.27)], respiratory, thoracic and mediastinal disorders [n=12,209, ROR (95% CI) = 1.57 (1.36-1.43)], Neoplasms benign, malignant and unspecified (including cysts and polyps) [n=10,924, ROR (95% CI) = 2.24(2.20-2.29)]. The absence of statistical significance for certain SOCs (e.g., congenital/familial/genetic disorders, surgical/medical procedures, social circumstances) was attributed to the lower bounds of their ROR 95% confidence intervals falling below 1.

**Table 2:** Signal strength of Nivolumab AEs across System Organ Classes (SOC) in the FAERS database.

System Organ Class (SOC)	Case numbers	ROR(95%CI)	PRR(χ^2^)	EBGM(EBGM05)	IC(IC025)
General disorders and administration site conditions	26822	0.85 (0.84 - 0.86)	0.87 (628.61)	0.87 (0.86)	-0.2 (-0.22)
Gastrointestinal disorders*	17549	1.25 (1.23 - 1.27)	1.22 (781.59)	1.22 (1.21)	0.29 (0.27)
Injury, poisoning and procedural complications	13009	0.66 (0.65 - 0.68)	0.69 (2029.64)	0.69 (0.68)	-0.53 (-0.56)
Respiratory, thoracic and mediastinal disorders*	12209	1.57 (1.54 - 1.6)	1.53 (2341.66)	1.53 (1.5)	0.61 (0.58)
Neoplasms benign, malignant and unspecified (incl cysts and polyps) *	10924	2.24 (2.2 - 2.29)	2.17 (6995.46)	2.15 (2.12)	1.11 (1.08)
Nervous system disorders	10102	0.72 (0.71 - 0.74)	0.74 (1008.98)	0.74 (0.73)	-0.43 (-0.46)
Infections and infestations*	9936	1.07 (1.05 - 1.09)	1.07 (42.05)	1.07 (1.05)	0.09 (0.06)
Investigations	9864	0.98 (0.96 - 1)	0.98 (2.68)	0.98 (0.97)	-0.02 (-0.05)
Skin and subcutaneous tissue disorders	9370	0.97 (0.95 - 0.99)	0.97 (11.13)	0.97 (0.95)	-0.05 (-0.08)
Musculoskeletal and connective tissue disorders	7678	0.85 (0.83 - 0.87)	0.86 (197.9)	0.86 (0.84)	-0.22 (-0.26)
Metabolism and nutrition disorders*	7414	2.17 (2.12 - 2.22)	2.11 (4408.3)	2.1 (2.06)	1.07 (1.04)
Endocrine disorders*	5387	13.18 (12.82 - 13.55)	12.8 (55634.95)	12.17 (11.89)	3.61 (3.56)
Hepatobiliary disorders*	5330	3.91 (3.8 - 4.02)	3.82 (11006.58)	3.77 (3.69)	1.92 (1.88)
Cardiac disorders*	5121	1.38 (1.34 - 1.42)	1.37 (515.87)	1.37 (1.33)	0.45 (0.41)
Blood and lymphatic system disorders*	4791	1.72 (1.67 - 1.77)	1.7 (1398.34)	1.7 (1.66)	0.76 (0.72)
Renal and urinary disorders*	4253	1.29 (1.25 - 1.33)	1.28 (269.82)	1.28 (1.25)	0.36 (0.31)
Vascular disorders	2867	0.85 (0.82 - 0.88)	0.85 (74.83)	0.85 (0.83)	-0.23 (-0.28)
Psychiatric disorders	2436	0.25 (0.24 - 0.26)	0.26 (5319.73)	0.26 (0.25)	-1.92 (-1.98)
Eye disorders	2347	0.68 (0.66 - 0.71)	0.69 (337.04)	0.69 (0.67)	-0.54 (-0.6)
Immune system disorders	1714	0.83 (0.79 - 0.87)	0.83 (57.22)	0.83 (0.8)	-0.26 (-0.33)
Surgical and medical procedures	1267	0.51 (0.48 - 0.54)	0.51 (593.32)	0.51 (0.49)	-0.96 (-1.04)
Ear and labyrinth disorders	441	0.59 (0.53 - 0.64)	0.59 (128.71)	0.59 (0.54)	-0.77 (-0.9)
Reproductive system and breast disorders	303	0.23 (0.21 - 0.26)	0.24 (756.24)	0.24 (0.22)	-2.08 (-2.25)
Product issues	142	0.04 (0.04 - 0.05)	0.05 (2877.52)	0.05 (0.04)	-4.44 (-4.69)
Social circumstances	106	0.14 (0.12 - 0.17)	0.14 (555.79)	0.14 (0.12)	-2.82 (-3.1)
Pregnancy, puerperium and perinatal conditions	92	0.14 (0.11 - 0.17)	0.14 (502.89)	0.14 (0.12)	-2.86 (-3.16)
Congenital, familial and genetic disorders	47	0.1 (0.07 - 0.13)	0.1 (388.29)	0.1 (0.08)	-3.34 (-3.75)

Asterisks (*) indicate statistically significant signals in algorithm; ROR, reporting odds ratio; PRR, proportional reporting ratio; EBGM, empirical Bayesian geometric mean; EBGM05, the lower limit of the 95% CI of EBGM; IC, information component; IC025, the lower limit of the 95% CI of the IC; CI, confidence interval; AEs, adverse events.

### 3.3 Signal detection related to PT levels

We systematically classified all nivolumab-associated AEs by frequency, with comprehensive results presented in **Table 3**. All AEs meeting the positive signal criteria were meticulously documented in **Supplementary Table S3**. The top 10 reported PTs included Death, Malignant neoplasm progression, Off label use, Intentional product use issue, Diarrhoea, Fatigue, Pyrexia, Dyspnoea, Rash and Nausea. Several PTs were consistent with those listed in the drug label, including fatigue, dyspnoea, musculoskeletal pain, decreased appetite, cough, nausea, and constipation. Notably, we identified potential adverse reactions not currently mentioned in the prescribing information, such as: Malignant neoplasm progression, Weight decreased, Sepsis, Myocarditis and Hypotension.

**Table 3:** Top 50 frequency of adverse events at the PT level for Nivolumab.

PT	Case numbers	ROR(95%CI)	PRR(χ^2^)	EBGM(EBGM05)	IC(IC025)
Death*	10018	4.22 (4.14 - 4.31)	4.04 (22821.49)	3.98 (3.92)	1.99 (1.96)
Malignant neoplasm progression*	7533	27.68 (27.01 - 28.36)	26.51 (166031.6)	23.86 (23.38)	4.58 (4.54)
Off label use*	3534	1.23 (1.19 - 1.27)	1.22 (146.72)	1.22 (1.19)	0.29 (0.24)
Intentional product use issue*	3093	10 (9.64 - 10.37)	9.84 (23591.12)	9.47 (9.19)	3.24 (3.19)
Diarrhoea*	2922	1.55 (1.5 - 1.61)	1.54 (560.99)	1.54 (1.49)	0.62 (0.57)
Fatigue*	2535	1.1 (1.06 - 1.14)	1.1 (21.47)	1.1 (1.06)	0.13 (0.07)
Pyrexia*	2213	2.42 (2.32 - 2.52)	2.4 (1795.9)	2.38 (2.3)	1.25 (1.19)
Dyspnoea*	1906	1.22 (1.17 - 1.28)	1.22 (74.65)	1.22 (1.17)	0.28 (0.22)
Rash*	1713	1.4 (1.33 - 1.47)	1.4 (191.95)	1.39 (1.34)	0.48 (0.41)
Nausea	1675	0.77 (0.74 - 0.81)	0.78 (108.71)	0.78 (0.75)	-0.36 (-0.43)
Decreased appetite*	1653	2.48 (2.36 - 2.6)	2.46 (1427.65)	2.45 (2.35)	1.29 (1.22)
Pneumonia*	1624	1.8 (1.71 - 1.89)	1.79 (565.18)	1.78 (1.71)	0.84 (0.76)
Colitis*	1418	14.17 (13.43 - 14.95)	14.06 (16216.66)	13.3 (12.72)	3.73 (3.65)
Asthenia*	1405	1.37 (1.3 - 1.45)	1.37 (140.12)	1.37 (1.31)	0.45 (0.37)
Pneumonitis*	1301	17.82 (16.84 - 18.86)	17.7 (19033.68)	16.5 (15.74)	4.04 (3.96)
Vomiting	1217	0.99 (0.94 - 1.05)	0.99 (0.13)	0.99 (0.94)	-0.01 (-0.1)
Weight decreased*	1168	1.49 (1.41 - 1.58)	1.49 (187.93)	1.49 (1.42)	0.57 (0.49)
Hypothyroidism*	1148	14.72 (13.86 - 15.62)	14.62 (13704.19)	13.81 (13.13)	3.79 (3.7)
Pruritus*	1100	1.07 (1.01 - 1.14)	1.07 (5.08)	1.07 (1.02)	0.1 (0.01)
Acute kidney injury*	1092	1.93 (1.82 - 2.05)	1.92 (479.91)	1.91 (1.82)	0.94 (0.85)
General physical health deterioration*	1074	3.56 (3.36 - 3.79)	3.55 (1939.04)	3.51 (3.34)	1.81 (1.72)
Anaemia*	1046	2.11 (1.98 - 2.24)	2.1 (599.31)	2.09 (1.99)	1.06 (0.97)
Adverse event*	1041	4.02 (3.78 - 4.27)	4 (2305.02)	3.95 (3.75)	1.98 (1.89)
Product use in unapproved indication*	1034	1.23 (1.16 - 1.31)	1.23 (44.57)	1.23 (1.17)	0.3 (0.21)
Arthralgia	1015	0.84 (0.79 - 0.89)	0.84 (30.85)	0.84 (0.8)	-0.25 (-0.34)
Malaise	904	0.69 (0.65 - 0.74)	0.7 (120.48)	0.7 (0.66)	-0.52 (-0.62)
Interstitial lung disease*	894	7 (6.55 - 7.48)	6.97 (4438.7)	6.79 (6.42)	2.76 (2.67)
Headache	876	0.49 (0.46 - 0.52)	0.49 (464.8)	0.49 (0.47)	-1.02 (-1.12)
Pain	875	0.47 (0.44 - 0.5)	0.47 (513.5)	0.48 (0.45)	-1.07 (-1.17)
Cough*	836	1.02 (0.95 - 1.09)	1.02 (0.28)	1.02 (0.96)	0.03 (-0.07)
Back pain*	819	1.25 (1.17 - 1.34)	1.25 (41.84)	1.25 (1.18)	0.32 (0.22)
Sepsis*	799	2.7 (2.52 - 2.9)	2.69 (842.69)	2.67 (2.52)	1.42 (1.32)
Constipation*	792	1.3 (1.21 - 1.39)	1.29 (52.89)	1.29 (1.22)	0.37 (0.27)
Pleural effusion*	709	4.64 (4.31 - 5)	4.63 (1977.32)	4.55 (4.28)	2.19 (2.08)
Adrenal insufficiency*	700	22.38 (20.71 - 24.19)	22.29 (12976.82)	20.41 (19.12)	4.35 (4.24)
Immune-mediated enterocolitis*	679	100.49 (91.81 - 109.98)	100.09 (46368.89)	69.98 (64.88)	6.13 (6)
Myocarditis*	656	22.68 (20.92 - 24.57)	22.59 (12325.25)	20.66 (19.31)	4.37 (4.25)
Dehydration*	654	1.97 (1.82 - 2.12)	1.96 (306.46)	1.95 (1.83)	0.97 (0.85)
Thrombocytopenia*	628	2.15 (1.99 - 2.33)	2.15 (383.4)	2.14 (2)	1.1 (0.98)
Hepatic function abnormal*	604	6.54 (6.03 - 7.1)	6.52 (2748.28)	6.37 (5.95)	2.67 (2.55)
Abdominal pain	600	0.98 (0.9 - 1.06)	0.98 (0.23)	0.98 (0.92)	-0.03 (-0.15)
Respiratory failure*	592	3.32 (3.06 - 3.6)	3.31 (941.9)	3.28 (3.06)	1.71 (1.59)
Fall	576	0.63 (0.58 - 0.68)	0.63 (127)	0.63 (0.59)	-0.67 (-0.79)
Hypotension*	572	1.06 (0.97 - 1.15)	1.06 (1.72)	1.06 (0.99)	0.08 (-0.04)
Drug ineffective	560	0.13 (0.12 - 0.15)	0.14 (3113.51)	0.14 (0.13)	-2.86 (-2.98)
Dizziness	547	0.4 (0.37 - 0.44)	0.4 (485.74)	0.4 (0.38)	-1.31 (-1.43)
Hyperthyroidism*	539	14.33 (13.13 - 15.63)	14.29 (6270.31)	13.51 (12.56)	3.76 (3.63)
Renal failure*	539	1.49 (1.37 - 1.62)	1.49 (85.95)	1.49 (1.38)	0.57 (0.45)
Product use issue	522	0.75 (0.69 - 0.82)	0.75 (42.34)	0.75 (0.7)	-0.41 (-0.53)
Liver disorder*	521	4.67 (4.28 - 5.09)	4.66 (1468.73)	4.59 (4.27)	2.2 (2.07)

Asterisks (*) indicate statistically significant signals in algorithm; ROR, reporting odds ratio; PRR, proportional reporting ratio; EBGM, empirical Bayesian geometric mean; EBGM05, the lower limit of the 95% CI of EBGM; IC, information component; IC025, the lower limit of the 95% CI of the IC; CI, confidence interval; PT, preferred term.

### 3.4 Information from subgroup

**Supplementary Tables S4**-**S11** present comprehensive subgroup analyses of nivolumab-associated adverse events. In the gender subgroup analysis, death was the most frequently reported adverse event in both males and females, while males exhibited a higher propensity for diarrhea and females demonstrated greater susceptibility to fatigue. Age-stratified analysis revealed that pyrexia was more commonly reported in individuals under 18 years old, whereas mortality rates showed a relative increase in patients aged 18 years and above. Healthcare professionals were more likely to report malignant neoplasm progression, reflecting their clinical awareness in cancer patient monitoring, while consumer reports predominantly involved fatal outcomes. These findings highlight distinct adverse event profiles across different demographic subgroups, with detailed statistical results including reporting odds ratios and confidence intervals provided in the **Supplementary Tables**.

### 3.5 Sensitivity analysis

Based on baseline data from the FAERS database, the primary indications for nivolumab were NSCLC and MM. Consequently, we excluded certain concomitant medications potentially used to treat these two malignancies, primarily ipilimumab. After excluding cases requiring additional combination therapies, a total of 48,923 case reports comprising 126,385 AEs were identified. The predominant reported adverse reactions included death, malignant neoplasm progression, off-label use, intentional product use issue, and diarrhea (see **Supplementary Table 12** for detailed information).

### 3.6 Time-to-onset and weibull distribution analysis of AEs based on nivolumab

**Figure 3:**
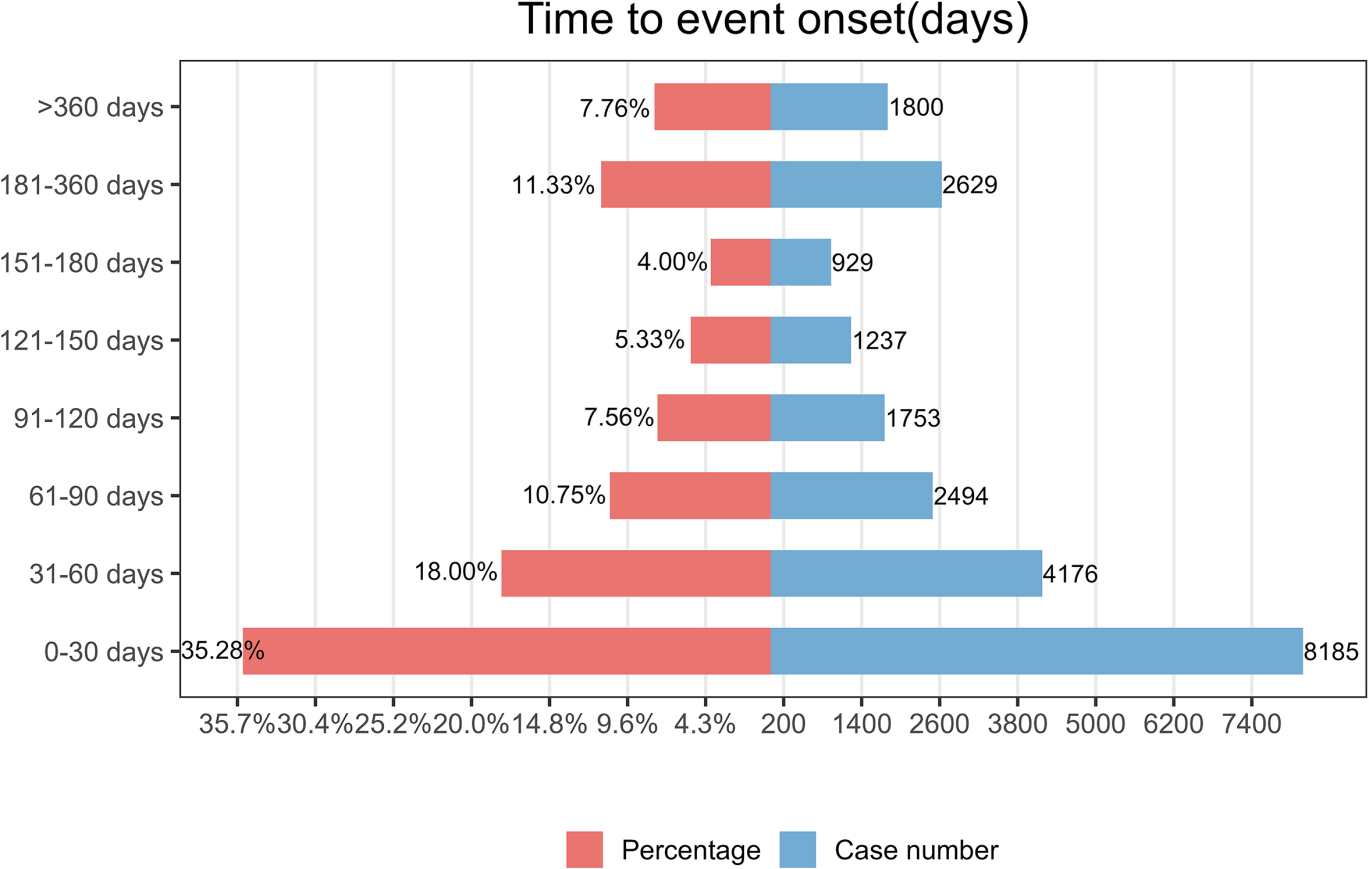
Time-to-onset of AEs.

As illustrated in **Figure 3**, the majority of nivolumab-associated AEs occurred within the initial 30-day period following treatment initiation. Furthermore, Weibull distribution analysis demonstrated an early failure pattern, with specific distribution parameters detailed in **Table 4**.

**Table 4:** Time to onset of Nivolumab-associated adverse events and Weibull distribution analysis.

Drug	TTO(days)		Weibull distribution		
	Case reports	Median(d)(IQR)	Scale parameter: α(95%CI)	Shape parameter: β(95%CI)	Type
Nivolumab	64627	55(20-139)	101.20(99.38-103.02)	0.76(0.75-0.76)	Early failure

TTO, time to onset; CI, confidence interval; IQR, interquartile range.

## 4 Discussion

The present study analyzed adverse events associated with nivolumab recorded in the FAERS database following its FDA approval and market introduction in the fourth quarter of 2014. These findings not only confirmed various adverse reactions previously documented in the prescribing information, including fatigue, dyspnea, musculoskeletal pain, decreased appetite, cough, nausea and constipation, but also identified additional adverse events not currently included in the labeling information, such as malignant neoplasm progression, weight loss, sepsis, myocarditis and hypotension.

The present study demonstrates partial concordance between the top 10 most frequently reported adverse drug reactions (ADRs) associated with nivolumab and those listed in the prescribing information, thereby corroborating that fatigue, nausea, and dyspnea represent common adverse effects of nivolumab. A meta-analysis has shown fatigue to be the most prevalent ADR induced by PD-1/PD-L1 inhibitors, consistently ranking first among both all-grade and grade ≥3 adverse events (22). The frequent reporting of these ADRs may be attributed to multiple factors: their characteristic clinical manifestations facilitate accurate recognition and documentation by healthcare providers, while concurrent non-drug-related symptoms might be erroneously ascribed to nivolumab therapy. The prolonged duration of these symptoms could reflect the sustained immunological activation underlying the drug’s antitumor mechanism, potentially leading to persistent or progressive immune-related adverse events over time (23). Notably, the prescribing information also highlights potentially fatal immune-mediated adverse reactions including pneumonitis, colitis, hepatitis, nephritis, and renal dysfunction, which demand heightened clinical vigilance across medical specialties. A comprehensive meta-analysis incorporating data from hundreds of clinical studies on fatal toxicities associated with immune checkpoint inhibitors (ICIs) revealed that immune-mediated colitis accounted for the highest number of reported mortality cases, followed by immune-mediated pneumonitis, hepatitis, and nephritis. Importantly, the diagnostic challenges posed by these rare but serious ADRs warrant emphasis - for instance, immune-mediated hepatitis may be clinically indistinguishable from hepatic dysfunction secondary to metastatic infiltration or perfusion abnormalities (resulting from distributive, hypovolemic, or cardiogenic shock) (24). Enhanced recognition of these ADRs is crucial for implementing timely preventive measures and therapeutic interventions, which are of paramount importance for cancer patients receiving immunotherapy.

Epidemiological studies demonstrate that the overall incidence rates of NSCLC and malignant melanoma follow similar age-related patterns as most malignancies, showing a progressive increase with advancing age (25, 26). Furthermore, male patients exhibit significantly higher susceptibility to both NSCLC and malignant melanoma compared to females. These epidemiological characteristics directly correlate with our baseline data, which revealed a substantially higher number of adverse event (AE) reports among male patients. Additionally, the 65–85 year age group showed the highest proportion of AE reports compared to other age strata. The United States accounted for the majority of reported cases, likely attributable to the domestic nature of the FAERS reporting system. The temporal analysis demonstrated a steady increase in nivolumab-associated AE reports from 2017 to 2019, reflecting its expanding clinical utilization. Notably, 2020 marked the first observed decline in reporting frequency, which may primarily reflect the impact of the COVID-19 pandemic on therapeutic administration patterns.

While nivolumab has demonstrated significant survival benefits in NSCLC and malignant melanoma, therapeutic response varies substantially among patients, resulting in malignant neoplasm progression emerging as the most frequently reported adverse event besides mortality. In the CheckMate 816 trial evaluating nivolumab plus platinum-based chemotherapy for NSCLC, disease progression rates were 23.9% at 1-year follow-up, increasing to 36.2% by 2 years (15). Similarly, the CheckMate 274 trial investigating nivolumab as adjuvant therapy for high-risk, muscle-invasive urothelial carcinoma post-radical surgery reported progression rates of 25.1% at 6 months, with 48.2% of patients experiencing tumor recurrence or death at median follow-up (approximately 20 months) (27).

Weight loss frequently co-occurs with tumor progression as a clinically significant paraneoplastic phenomenon. These findings strongly suggest that such treatment-related outcomes, including both malignant neoplasm progression and associated weight loss, warrant explicit inclusion in the prescribing information to enhance clinical recognition and management.

Myocarditis, encephalitis, and sepsis constitute severe treatment-related complications that may directly endanger patients’ lives, warranting particular clinical attention. In the RELATIVITY-047 clinical study, 0.6% (2 cases) of patients receiving nivolumab monotherapy developed myocarditis, including one fatal case complicated by concurrent myocarditis and septicemia (15). The CheckMate 451 study demonstrated that among 278 patients treated with nivolumab plus ipilimumab, seven experienced fatal adverse drug reactions, including one case each of myocarditis and sepsis with end-organ failure, while one case of fatal encephalitis occurred among 279 patients receiving nivolumab monotherapy (28). A study analyzing two fatal myocarditis cases in malignant melanoma patients treated with nivolumab-ipilimumab combination therapy suggested that compared with nivolumab monotherapy, the combination regimen may lead to more frequent and severe myocarditis (29). Animal studies have confirmed that PD-1 plays a regulatory role in T-cell-mediated myocardial immune responses in experimental myocarditis models, potentially preventing inflammation and myocyte damage (30). PD-1 gene deficiency in mice can also induce cardiomyopathy triggered by anti-cardiac troponin I autoantibodies (31, 32). Furthermore, cases of immunotherapy-associated encephalitis followed by interstitial granulomatous dermatitis have been reported in metastatic melanoma patients treated with nivolumab and ipilimumab (33). Regarding sepsis, one clinical trial reported two fatal cases among 361 patients receiving nivolumab-ipilimumab combination therapy (34), with similar cases documented in detailed reports (35).

Hypotension represents a frequently observed yet currently unlisted adverse effect in nivolumab’s prescribing information. A clinical trial investigating nivolumab for metastatic sarcoma reported four cases of hypotension occurring during combination therapy with ipilimumab (36). Beyond direct causation, nivolumab may induce hypotension through immune-related adrenal insufficiency, as evidenced by a documented case of refractory hypotension secondary to nivolumab-induced adrenal crisis in a 52-year-old male patient (37). Furthermore, nivolumab can trigger autoimmune autonomic ganglionopathy, potentially leading to severe orthostatic hypotension resistant to conventional management. The underlying pathophysiology may involve autoreactive T-cell activation, as PD-1/CTLA-4 blockade disrupts critical self-tolerance mechanisms, precipitating various autoimmune manifestations. Alternative hypotheses suggest B-cell mediated autoantibody production via T-cell dependent activation. Additional proposed mechanisms of immune checkpoint inhibitor neurotoxicity include inflammatory processes affecting neural microstructures, such as endoneurial microvilli inflammation and subperineurial inflammatory edema (38). These findings underscore the necessity for comprehensive monitoring beyond symptomatic management of immune-related adverse events during nivolumab therapy. Elucidation of the multifactorial pathogenic mechanisms will facilitate more effective management of these complex pharmacological complications.

This study has several limitations that warrant consideration. First, the FAERS database relies primarily on voluntary reporting by healthcare professionals and consumers, which may introduce selection bias through underreporting, duplicate entries, and inaccurate documentation. Second, the database lacks standardized classification of adverse event (AE) severity and does not distinguish between drug-induced AEs and those attributable to underlying comorbidities or concomitant therapies. This limitation creates challenges in determining AE causality and grading, as disease-related symptoms may be inadvertently reported as treatment-emergent AEs. Consequently, clinical judgment remains essential for accurate AE assessment in practice. Third, the FAERS data reflect only immediate post-treatment outcomes without longitudinal follow-up information, limiting the ability to evaluate long-term drug safety profiles in real-world settings. Furthermore, the database exhibits significant geographical bias, with predominant representation of U.S. case reports, thereby constraining the generalizability of findings to global populations and diverse disease contexts. These inherent constraints emphasize the need for complementary prospective studies to validate the pharmacovigilance signals identified in this analysis.

## 5 Conclusion

This pharmacovigilance study utilized the FAERS database to evaluate the real-world safety profile of nivolumab. The analysis confirmed frequently reported AEs consistent with the drug label, including fatigue, dyspnea, musculoskeletal pain, decreased appetite, cough, nausea, and constipation. Importantly, we identified several clinically significant AEs not currently listed in the prescribing information: malignant neoplasm progression, weight loss, sepsis, myocarditis, encephalitis, and hypotension. These findings provide healthcare professionals with enhanced awareness of potential nivolumab-related toxicities, enabling more comprehensive risk-benefit assessments and improved clinical management of treated patients. The detection of these safety signals underscores the value of post-marketing surveillance in complementing data from controlled clinical trials.

## Data Availability

Publicly available datasets were analyzed in this study. This data can be found here: https://fis.fda.gov/extensions/FPD-QDE-FAERS/FPD-QDE-FAERS.html.
