# Supplementary material for: The real-world safety of Nivolumab: a pharmacovigilance analysis based on the FDA adverse event reporting system
Source: Front Immunol. 2025 May 26;16:1605958. doi: 10.3389/fimmu.2025.1605958 (PMC12146392; doi:10.3389/fimmu.2025.1605958)
Supplement: Supplementary file 1 [file Table1.docx]

Supplementary Material

Supplementary Tables

Supplementary Table 1:

Two-by-two contingency table for disproportionality analyses.

|  | Target AEs | Other AEs | Total |
| --- | --- | --- | --- |
|  | a | b | a+b |
| Other drugs | c | d | c+d |
| Total | a+c | b+d | a+b+c+d |

Abbreviation: AEs, adverse events; a, number of reports containing both the target drug and target adverse drug reaction; b, number of reports containing other adverse drug reaction of the target drug; c, number of reports containing the target adverse drug reaction of other drugs; d, number of reports containing other drugs and other adverse drug reactions.

Supplementary Table 2:

Four major algorithms used for signal detection.

| Algorithms | Equation | Criteria |
| --- | --- | --- |
| ROR | ROR=ad/b/c | lower limit of 95% CI>1, N≥3 |
|  | 95%CI=e^ln(ROR)±1.96(1/a+1/b+1/c+1/d)^0.5^ |  |
| PRR | PRR=a(c+d)/c/(a+b) | PRR≥2, χ^2^≥4, N≥3 |
|  | χ^2^=[(ad-bc)^2](a+b+c+d)/[(a+b)(c+d)(a+c)(b+d)] |  |
| BCPNN | IC=log_2_a(a+b+c+d)(a+c)(a+b) | IC025>0 |
|  | 95%CI= E(IC) ± 2V(IC)^0.5 |  |
| MGPS | EBGM=a(a+b+c+d)/(a+c)/(a+b) | EBGM05>2 |
|  | 95%CI=e^ln(EBGM)±1.96(1/a+1/b+1/c+1/d)^0.5^ |  |

Abbreviation: a, number of reports containing both the target drug and target adverse drug reaction; b, number of reports containing other adverse drug reaction of the target drug; c, number of reports containing the target adverse drug reaction of other drugs; d, number of reports containing other drugs and other adverse drug reactions. 95%CI, 95% confidence interval; N, the number of reports; χ2, chi-squared; IC, information component; IC025, the lower limit of 95% CI of the IC; E(IC), the IC expectations; V(IC), the variance of IC; EBGM, empirical Bayesian geometric mean; EBGM05, the lower limit of 95% CI of EBGM.

Supplementary Table 3 :

Top 50 most frequent adverse events meeting the positive signal threshold at the PT level from FAERS data

| PT | Case numbers | ROR(95%CI) | PRR(χ^2^) | EBGM(EBGM05) | IC(IC025) |
| --- | --- | --- | --- | --- | --- |
| Death | 10018 | 4.22 ( 4.14 - 4.31 ) | 4.04 ( 22821.49 ) | 3.98 ( 3.92 ) | 1.99 ( 1.96 ) |
| Malignant neoplasm progression | 7533 | 27.68 ( 27.01 - 28.36 ) | 26.51 ( 166031.6 ) | 23.86 ( 23.38 ) | 4.58 ( 4.54 ) |
| Intentional product use issue | 3093 | 10 ( 9.64 - 10.37 ) | 9.84 ( 23591.12 ) | 9.47 ( 9.19 ) | 3.24 ( 3.19 ) |
| Pyrexia | 2213 | 2.42 ( 2.32 - 2.52 ) | 2.4 ( 1795.9 ) | 2.38 ( 2.3 ) | 1.25 ( 1.19 ) |
| Decreased appetite | 1653 | 2.48 ( 2.36 - 2.6 ) | 2.46 ( 1427.65 ) | 2.45 ( 2.35 ) | 1.29 ( 1.22 ) |
| Colitis | 1418 | 14.17 ( 13.43 - 14.95 ) | 14.06 ( 16216.66 ) | 13.3 ( 12.72 ) | 3.73 ( 3.65 ) |
| Pneumonitis | 1301 | 17.82 ( 16.84 - 18.86 ) | 17.7 ( 19033.68 ) | 16.5 ( 15.74 ) | 4.04 ( 3.96 ) |
| Hypothyroidism | 1148 | 14.72 ( 13.86 - 15.62 ) | 14.62 ( 13704.19 ) | 13.81 ( 13.13 ) | 3.79 ( 3.7 ) |
| General physical health deterioration | 1074 | 3.56 ( 3.36 - 3.79 ) | 3.55 ( 1939.04 ) | 3.51 ( 3.34 ) | 1.81 ( 1.72 ) |
| Adverse event | 1041 | 4.02 ( 3.78 - 4.27 ) | 4 ( 2305.02 ) | 3.95 ( 3.75 ) | 1.98 ( 1.89 ) |
| Interstitial lung disease | 894 | 7 ( 6.55 - 7.48 ) | 6.97 ( 4438.7 ) | 6.79 ( 6.42 ) | 2.76 ( 2.67 ) |
| Sepsis | 799 | 2.7 ( 2.52 - 2.9 ) | 2.69 ( 842.69 ) | 2.67 ( 2.52 ) | 1.42 ( 1.32 ) |
| Pleural effusion | 709 | 4.64 ( 4.31 - 5 ) | 4.63 ( 1977.32 ) | 4.55 ( 4.28 ) | 2.19 ( 2.08 ) |
| Adrenal insufficiency | 700 | 22.38 ( 20.71 - 24.19 ) | 22.29 ( 12976.82 ) | 20.41 ( 19.12 ) | 4.35 ( 4.24 ) |
| Immune-mediated enterocolitis | 679 | 100.49 ( 91.81 - 109.98 ) | 100.09 ( 46368.89 ) | 69.98 ( 64.88 ) | 6.13 ( 6 ) |
| Myocarditis | 656 | 22.68 ( 20.92 - 24.57 ) | 22.59 ( 12325.25 ) | 20.66 ( 19.31 ) | 4.37 ( 4.25 ) |
| Hepatic function abnormal | 604 | 6.54 ( 6.03 - 7.1 ) | 6.52 ( 2748.28 ) | 6.37 ( 5.95 ) | 2.67 ( 2.55 ) |
| Respiratory failure | 592 | 3.32 ( 3.06 - 3.6 ) | 3.31 ( 941.9 ) | 3.28 ( 3.06 ) | 1.71 ( 1.59 ) |
| Hyperthyroidism | 539 | 14.33 ( 13.13 - 15.63 ) | 14.29 ( 6270.31 ) | 13.51 ( 12.56 ) | 3.76 ( 3.63 ) |
| Pemphigoid | 521 | 26.84 ( 24.51 - 29.4 ) | 26.77 ( 11572.65 ) | 24.07 ( 22.31 ) | 4.59 ( 4.46 ) |
| Liver disorder | 521 | 4.67 ( 4.28 - 5.09 ) | 4.66 ( 1468.73 ) | 4.59 ( 4.27 ) | 2.2 ( 2.07 ) |
| Hepatitis | 517 | 8.73 ( 7.99 - 9.53 ) | 8.7 ( 3396.6 ) | 8.42 ( 7.82 ) | 3.07 ( 2.94 ) |
| Prescribed underdose | 501 | 7.29 ( 6.67 - 7.97 ) | 7.27 ( 2626.53 ) | 7.08 ( 6.57 ) | 2.82 ( 2.69 ) |
| Infusion related reaction | 497 | 2.68 ( 2.45 - 2.92 ) | 2.67 ( 514.22 ) | 2.65 ( 2.46 ) | 1.41 ( 1.28 ) |
| Pulmonary embolism | 477 | 2.3 ( 2.1 - 2.52 ) | 2.3 ( 345.57 ) | 2.28 ( 2.12 ) | 1.19 ( 1.06 ) |
| Lung disorder | 476 | 3.57 ( 3.26 - 3.91 ) | 3.56 ( 863.38 ) | 3.52 ( 3.26 ) | 1.82 ( 1.68 ) |
| Hyponatraemia | 473 | 3.27 ( 2.99 - 3.59 ) | 3.27 ( 734.94 ) | 3.24 ( 3 ) | 1.69 ( 1.56 ) |
| Autoimmune disorder | 458 | 18.64 ( 16.95 - 20.51 ) | 18.59 ( 7054.01 ) | 17.27 ( 15.95 ) | 4.11 ( 3.97 ) |
| Aspartate aminotransferase increased | 444 | 4.08 ( 3.71 - 4.48 ) | 4.07 ( 1011.19 ) | 4.02 ( 3.71 ) | 2.01 ( 1.87 ) |
| Hypophysitis | 438 | 83.32 ( 74.69 - 92.96 ) | 83.11 ( 26078.92 ) | 61.26 ( 55.9 ) | 5.94 ( 5.78 ) |
| Febrile neutropenia | 438 | 2.38 ( 2.16 - 2.61 ) | 2.37 ( 344.26 ) | 2.36 ( 2.18 ) | 1.24 ( 1.1 ) |
| Alanine aminotransferase increased | 437 | 3.3 ( 3 - 3.63 ) | 3.29 ( 688.98 ) | 3.26 ( 3.01 ) | 1.71 ( 1.57 ) |
| Myositis | 424 | 22.06 ( 19.96 - 24.37 ) | 22 ( 7757.43 ) | 20.16 ( 18.55 ) | 4.33 ( 4.19 ) |
| Diabetic ketoacidosis | 419 | 5.75 ( 5.22 - 6.34 ) | 5.74 ( 1600.4 ) | 5.62 ( 5.18 ) | 2.49 ( 2.35 ) |
| Stomatitis | 399 | 2.24 ( 2.03 - 2.48 ) | 2.24 ( 271.47 ) | 2.23 ( 2.05 ) | 1.16 ( 1.01 ) |
| Fulminant type 1 diabetes mellitus | 372 | 206.92 ( 179.85 - 238.06 ) | 206.47 ( 40019.8 ) | 109.1 ( 97.03 ) | 6.77 ( 6.59 ) |
| Type 1 diabetes mellitus | 369 | 26.98 ( 24.22 - 30.05 ) | 26.92 ( 8242.3 ) | 24.2 ( 22.11 ) | 4.6 ( 4.44 ) |
| Ascites | 347 | 4.57 ( 4.11 - 5.08 ) | 4.56 ( 945.9 ) | 4.49 ( 4.11 ) | 2.17 ( 2.01 ) |
| Myasthenia gravis | 346 | 16.95 ( 15.2 - 18.91 ) | 16.92 ( 4826.6 ) | 15.82 ( 14.44 ) | 3.98 ( 3.82 ) |
| Metastases to central nervous system | 342 | 10.06 ( 9.02 - 11.21 ) | 10.04 ( 2666.82 ) | 9.66 ( 8.82 ) | 3.27 ( 3.11 ) |
| Pericardial effusion | 336 | 5.75 ( 5.16 - 6.41 ) | 5.74 ( 1282.86 ) | 5.62 ( 5.13 ) | 2.49 ( 2.33 ) |
| Hypokalaemia | 330 | 2.79 ( 2.51 - 3.11 ) | 2.79 ( 374.59 ) | 2.77 ( 2.53 ) | 1.47 ( 1.31 ) |
| Immune-mediated hepatitis | 321 | 76.23 ( 67.19 - 86.5 ) | 76.09 ( 17860.15 ) | 57.38 ( 51.62 ) | 5.84 ( 5.66 ) |
| Autoimmune hepatitis | 318 | 18.19 ( 16.23 - 20.39 ) | 18.16 ( 4778.41 ) | 16.9 ( 15.36 ) | 4.08 ( 3.91 ) |
| Enterocolitis | 317 | 21.69 ( 19.33 - 24.34 ) | 21.65 ( 5706.03 ) | 19.87 ( 18.04 ) | 4.31 ( 4.14 ) |
| Thyroid disorder | 317 | 7.64 ( 6.83 - 8.55 ) | 7.63 ( 1768.48 ) | 7.42 ( 6.75 ) | 2.89 ( 2.73 ) |
| Immune-mediated lung disease | 314 | 108.11 ( 94.53 - 123.64 ) | 107.91 ( 22614.22 ) | 73.69 ( 65.86 ) | 6.2 ( 6.02 ) |
| Prescribed overdose | 301 | 5.19 ( 4.63 - 5.82 ) | 5.18 ( 994.5 ) | 5.09 ( 4.63 ) | 2.35 ( 2.18 ) |
| Hyperglycaemia | 292 | 3.28 ( 2.92 - 3.68 ) | 3.28 ( 455.37 ) | 3.24 ( 2.94 ) | 1.7 ( 1.53 ) |
| Thyroiditis | 290 | 38.16 ( 33.7 - 43.21 ) | 38.1 ( 8982.89 ) | 32.81 ( 29.57 ) | 5.04 ( 4.86 ) |

Abbreviation: ROR, reporting odds ratio; PRR, proportional reporting ratio; EBGM, empirical Bayesian geometric mean; EBGM05, the lower limit of the 95% CI of EBGM; IC, information component; IC025, the lower limit of the 95% CI of the IC; CI, confidence interval; PT,preferred term.

Supplementary Table 4:

Top 50 most frequent adverse events for Nivolumab at the preferred term (PT) level in males from FAERS data

| PT | Case numbers | ROR(95%CI) | PRR(χ^2^) | EBGM(EBGM05) | IC(IC025) |
| --- | --- | --- | --- | --- | --- |
| Death* | 5485 | 2.84 ( 2.76 - 2.92 ) | 2.74 ( 6067.61 ) | 2.71 ( 2.64 ) | 1.44 ( 1.4 ) |
| Malignant neoplasm progression* | 4658 | 22.51 ( 21.81 - 23.24 ) | 21.53 ( 78256.16 ) | 18.58 ( 18.09 ) | 4.22 ( 4.17 ) |
| Off label use* | 2088 | 1.24 ( 1.19 - 1.29 ) | 1.23 ( 92.65 ) | 1.23 ( 1.19 ) | 0.3 ( 0.24 ) |
| Diarrhoea* | 1819 | 1.67 ( 1.59 - 1.75 ) | 1.66 ( 471.44 ) | 1.65 ( 1.58 ) | 0.72 ( 0.65 ) |
| Intentional product use issue* | 1656 | 9.51 ( 9.04 - 10 ) | 9.37 ( 11563.13 ) | 8.8 ( 8.44 ) | 3.14 ( 3.06 ) |
| Fatigue* | 1546 | 1.2 ( 1.14 - 1.27 ) | 1.2 ( 51.79 ) | 1.2 ( 1.15 ) | 0.26 ( 0.19 ) |
| Pyrexia* | 1399 | 2.26 ( 2.15 - 2.39 ) | 2.25 ( 955.35 ) | 2.22 ( 2.13 ) | 1.15 ( 1.07 ) |
| Dyspnoea* | 1178 | 1.26 ( 1.19 - 1.34 ) | 1.26 ( 63.93 ) | 1.26 ( 1.2 ) | 0.33 ( 0.25 ) |
| Pneumonia* | 1076 | 1.71 ( 1.61 - 1.81 ) | 1.7 ( 308.44 ) | 1.69 ( 1.61 ) | 0.76 ( 0.67 ) |
| Decreased appetite* | 1075 | 2.41 ( 2.27 - 2.57 ) | 2.4 ( 865.17 ) | 2.37 ( 2.26 ) | 1.25 ( 1.16 ) |
| Rash* | 977 | 1.45 ( 1.36 - 1.54 ) | 1.44 ( 132.99 ) | 1.44 ( 1.37 ) | 0.53 ( 0.43 ) |
| Nausea | 881 | 0.94 ( 0.88 - 1 ) | 0.94 ( 3.76 ) | 0.94 ( 0.89 ) | -0.09 ( -0.19 ) |
| Asthenia* | 880 | 1.34 ( 1.26 - 1.43 ) | 1.34 ( 75.11 ) | 1.34 ( 1.26 ) | 0.42 ( 0.32 ) |
| Weight decreased* | 778 | 1.46 ( 1.36 - 1.57 ) | 1.46 ( 110.45 ) | 1.45 ( 1.37 ) | 0.54 ( 0.43 ) |
| Colitis* | 777 | 12.12 ( 11.26 - 13.05 ) | 12.04 ( 7193.71 ) | 11.09 ( 10.43 ) | 3.47 ( 3.36 ) |
| Pneumonitis* | 770 | 15.45 ( 14.33 - 16.65 ) | 15.34 ( 9223.78 ) | 13.81 ( 12.97 ) | 3.79 ( 3.68 ) |
| Acute kidney injury* | 742 | 1.64 ( 1.53 - 1.77 ) | 1.64 ( 182.25 ) | 1.63 ( 1.53 ) | 0.7 ( 0.6 ) |
| General physical health deterioration* | 719 | 3.38 ( 3.14 - 3.64 ) | 3.36 ( 1166.37 ) | 3.3 ( 3.1 ) | 1.72 ( 1.61 ) |
| Hypothyroidism* | 683 | 17.17 ( 15.85 - 18.6 ) | 17.06 ( 9116.8 ) | 15.17 ( 14.19 ) | 3.92 ( 3.81 ) |
| Interstitial lung disease* | 672 | 6.71 ( 6.21 - 7.26 ) | 6.68 ( 3085.58 ) | 6.39 ( 5.99 ) | 2.68 ( 2.56 ) |
| Pruritus* | 662 | 1.27 ( 1.18 - 1.37 ) | 1.27 ( 37.33 ) | 1.27 ( 1.19 ) | 0.34 ( 0.23 ) |
| Vomiting* | 641 | 1.08 ( 1 - 1.17 ) | 1.08 ( 3.68 ) | 1.08 ( 1.01 ) | 0.11 ( -0.01 ) |
| Anaemia* | 635 | 1.98 ( 1.83 - 2.15 ) | 1.98 ( 303.43 ) | 1.96 ( 1.84 ) | 0.97 ( 0.86 ) |
| Arthralgia* | 579 | 1.01 ( 0.93 - 1.09 ) | 1.01 ( 0.02 ) | 1.01 ( 0.94 ) | 0.01 ( -0.11 ) |
| Malaise | 549 | 0.85 ( 0.78 - 0.92 ) | 0.85 ( 14.88 ) | 0.85 ( 0.79 ) | -0.24 ( -0.36 ) |
| Cough* | 510 | 1.11 ( 1.01 - 1.21 ) | 1.11 ( 5.26 ) | 1.11 ( 1.03 ) | 0.15 ( 0.02 ) |
| Pain | 504 | 0.5 ( 0.46 - 0.55 ) | 0.51 ( 242.51 ) | 0.51 ( 0.47 ) | -0.97 ( -1.1 ) |
| Product use in unapproved indication | 493 | 0.99 ( 0.91 - 1.08 ) | 0.99 ( 0.05 ) | 0.99 ( 0.92 ) | -0.01 ( -0.15 ) |
| Constipation* | 482 | 1.35 ( 1.24 - 1.48 ) | 1.35 ( 43.92 ) | 1.35 ( 1.25 ) | 0.43 ( 0.3 ) |
| Sepsis* | 482 | 2.12 ( 1.94 - 2.33 ) | 2.12 ( 281 ) | 2.1 ( 1.95 ) | 1.07 ( 0.94 ) |
| Adrenal insufficiency* | 467 | 21.49 ( 19.48 - 23.71 ) | 21.4 ( 7782.8 ) | 18.48 ( 17.02 ) | 4.21 ( 4.06 ) |
| Headache | 448 | 0.57 ( 0.52 - 0.63 ) | 0.58 ( 140.55 ) | 0.58 ( 0.53 ) | -0.79 ( -0.93 ) |
| Back pain* | 441 | 1.26 ( 1.15 - 1.39 ) | 1.26 ( 23.68 ) | 1.26 ( 1.16 ) | 0.33 ( 0.19 ) |
| Adverse event* | 436 | 3.21 ( 2.92 - 3.53 ) | 3.2 ( 645.2 ) | 3.15 ( 2.91 ) | 1.65 ( 1.51 ) |
| Pleural effusion* | 431 | 3.76 ( 3.42 - 4.14 ) | 3.75 ( 845.68 ) | 3.67 ( 3.39 ) | 1.88 ( 1.74 ) |
| Dehydration* | 420 | 1.92 ( 1.75 - 2.12 ) | 1.92 ( 182.82 ) | 1.91 ( 1.76 ) | 0.93 ( 0.79 ) |
| Myocarditis* | 396 | 15.01 ( 13.53 - 16.67 ) | 14.96 ( 4620.54 ) | 13.5 ( 12.37 ) | 3.75 ( 3.6 ) |
| Immune-mediated enterocolitis* | 380 | 60.3 ( 53.38 - 68.13 ) | 60.08 ( 15029 ) | 41.22 ( 37.22 ) | 5.37 ( 5.2 ) |
| Hepatic function abnormal* | 379 | 4.93 ( 4.45 - 5.47 ) | 4.92 ( 1140.91 ) | 4.78 ( 4.38 ) | 2.26 ( 2.1 ) |
| Respiratory failure* | 372 | 2.52 ( 2.27 - 2.79 ) | 2.51 ( 332.68 ) | 2.48 ( 2.28 ) | 1.31 ( 1.16 ) |
| Thrombocytopenia* | 369 | 1.74 ( 1.57 - 1.93 ) | 1.74 ( 113.8 ) | 1.73 ( 1.58 ) | 0.79 ( 0.64 ) |
| Renal failure* | 365 | 1.23 ( 1.11 - 1.37 ) | 1.23 ( 15.92 ) | 1.23 ( 1.13 ) | 0.3 ( 0.15 ) |
| Pemphigoid* | 364 | 21.32 ( 19.08 - 23.83 ) | 21.25 ( 6025.08 ) | 18.37 ( 16.74 ) | 4.2 ( 4.04 ) |
| Fall | 348 | 0.65 ( 0.58 - 0.72 ) | 0.65 ( 65.68 ) | 0.65 ( 0.6 ) | -0.62 ( -0.77 ) |
| Hypotension | 344 | 0.88 ( 0.79 - 0.98 ) | 0.88 ( 5.31 ) | 0.88 ( 0.81 ) | -0.18 ( -0.33 ) |
| Lung disorder* | 340 | 3.9 ( 3.5 - 4.35 ) | 3.9 ( 710.67 ) | 3.81 ( 3.48 ) | 1.93 ( 1.77 ) |
| Abdominal pain | 330 | 0.98 ( 0.88 - 1.09 ) | 0.98 ( 0.16 ) | 0.98 ( 0.89 ) | -0.03 ( -0.19 ) |
| Atrial fibrillation* | 325 | 1.53 ( 1.37 - 1.71 ) | 1.53 ( 58.9 ) | 1.52 ( 1.39 ) | 0.61 ( 0.45 ) |
| Dizziness | 316 | 0.43 ( 0.39 - 0.48 ) | 0.43 ( 236.66 ) | 0.43 ( 0.4 ) | -1.2 ( -1.37 ) |
| Prescribed underdose* | 311 | 7.07 ( 6.31 - 7.93 ) | 7.05 ( 1532.02 ) | 6.74 ( 6.12 ) | 2.75 ( 2.58 ) |

Abbreviation: Asterisks (*) indicate statistically significant signals in algorithm; ROR, reporting odds ratio; PRR, proportional reporting ratio; EBGM, empirical Bayesian geometric mean; EBGM05, the lower limit of the 95% CI of EBGM; IC, information component; IC025, the lower limit of the 95% CI of the IC; CI, confidence interval; PT,preferred term; AEs, adverse events.

Supplementary Table 5:

Top 50 most frequent adverse events for Nivolumab at the PT level in females from FAERS data

| PT | Case numbers | ROR(95%CI) | PRR(χ^2^) | EBGM(EBGM05) | IC(IC025) |
| --- | --- | --- | --- | --- | --- |
| Death* | 2997 | 5.4 ( 5.21 - 5.61 ) | 5.17 ( 10052.57 ) | 5.12 ( 4.96 ) | 2.35 ( 2.3 ) |
| Malignant neoplasm progression* | 2389 | 32.12 ( 30.78 - 33.51 ) | 30.81 ( 63870.12 ) | 28.59 ( 27.59 ) | 4.84 ( 4.78 ) |
| Intentional product use issue* | 1313 | 15.98 ( 15.11 - 16.9 ) | 15.64 ( 17310.2 ) | 15.06 ( 14.38 ) | 3.91 ( 3.83 ) |
| Off label use* | 1293 | 1.5 ( 1.42 - 1.58 ) | 1.49 ( 209.56 ) | 1.49 ( 1.42 ) | 0.57 ( 0.49 ) |
| Diarrhoea* | 962 | 1.46 ( 1.37 - 1.55 ) | 1.45 ( 135.71 ) | 1.45 ( 1.37 ) | 0.53 ( 0.44 ) |
| Fatigue* | 897 | 1.09 ( 1.02 - 1.16 ) | 1.09 ( 6.5 ) | 1.09 ( 1.03 ) | 0.12 ( 0.02 ) |
| Nausea | 741 | 0.87 ( 0.81 - 0.93 ) | 0.87 ( 14.79 ) | 0.87 ( 0.82 ) | -0.2 ( -0.31 ) |
| Pyrexia* | 696 | 2.41 ( 2.23 - 2.59 ) | 2.39 ( 561.75 ) | 2.38 ( 2.24 ) | 1.25 ( 1.14 ) |
| Dyspnoea* | 644 | 1.19 ( 1.1 - 1.29 ) | 1.19 ( 19.42 ) | 1.19 ( 1.11 ) | 0.25 ( 0.13 ) |
| Rash* | 591 | 1.37 ( 1.26 - 1.49 ) | 1.37 ( 58.3 ) | 1.37 ( 1.28 ) | 0.45 ( 0.33 ) |
| Decreased appetite* | 537 | 2.5 ( 2.3 - 2.73 ) | 2.49 ( 477.72 ) | 2.48 ( 2.31 ) | 1.31 ( 1.19 ) |
| Vomiting* | 535 | 1.16 ( 1.06 - 1.26 ) | 1.15 ( 11.12 ) | 1.15 ( 1.07 ) | 0.21 ( 0.08 ) |
| Asthenia* | 478 | 1.39 ( 1.27 - 1.52 ) | 1.39 ( 51.68 ) | 1.39 ( 1.28 ) | 0.47 ( 0.34 ) |
| Colitis* | 474 | 15.02 ( 13.7 - 16.47 ) | 14.91 ( 5922.35 ) | 14.39 ( 13.32 ) | 3.85 ( 3.71 ) |
| Pneumonia* | 471 | 1.68 ( 1.53 - 1.84 ) | 1.67 ( 127.37 ) | 1.67 ( 1.55 ) | 0.74 ( 0.61 ) |
| Headache | 401 | 0.56 ( 0.51 - 0.62 ) | 0.56 ( 139.52 ) | 0.56 ( 0.52 ) | -0.83 ( -0.98 ) |
| Hypothyroidism* | 400 | 13.9 ( 12.57 - 15.36 ) | 13.81 ( 4588.03 ) | 13.36 ( 12.29 ) | 3.74 ( 3.59 ) |
| Pneumonitis* | 380 | 18.64 ( 16.81 - 20.66 ) | 18.52 ( 6009.13 ) | 17.71 ( 16.24 ) | 4.15 ( 3.99 ) |
| Arthralgia | 378 | 0.81 ( 0.73 - 0.89 ) | 0.81 ( 17.29 ) | 0.81 ( 0.74 ) | -0.31 ( -0.46 ) |
| Pruritus | 374 | 0.98 ( 0.88 - 1.08 ) | 0.98 ( 0.17 ) | 0.98 ( 0.9 ) | -0.03 ( -0.18 ) |
| Anaemia* | 371 | 2.47 ( 2.23 - 2.74 ) | 2.46 ( 321.62 ) | 2.46 ( 2.25 ) | 1.3 ( 1.15 ) |
| Weight decreased* | 370 | 1.5 ( 1.35 - 1.66 ) | 1.5 ( 60.99 ) | 1.49 ( 1.37 ) | 0.58 ( 0.43 ) |
| Product use in unapproved indication* | 366 | 1.53 ( 1.38 - 1.7 ) | 1.53 ( 66.44 ) | 1.52 ( 1.4 ) | 0.61 ( 0.46 ) |
| Pain | 337 | 0.49 ( 0.44 - 0.55 ) | 0.5 ( 175.45 ) | 0.5 ( 0.45 ) | -1.01 ( -1.17 ) |
| Acute kidney injury* | 314 | 2.4 ( 2.15 - 2.68 ) | 2.39 ( 253.42 ) | 2.38 ( 2.17 ) | 1.25 ( 1.09 ) |
| Back pain* | 312 | 1.27 ( 1.14 - 1.42 ) | 1.27 ( 17.78 ) | 1.27 ( 1.15 ) | 0.34 ( 0.18 ) |
| General physical health deterioration* | 311 | 3.45 ( 3.08 - 3.86 ) | 3.44 ( 533.46 ) | 3.42 ( 3.11 ) | 1.77 ( 1.61 ) |
| Adverse event* | 310 | 4.08 ( 3.65 - 4.57 ) | 4.07 ( 710.74 ) | 4.04 ( 3.67 ) | 2.01 ( 1.85 ) |
| Malaise | 309 | 0.62 ( 0.55 - 0.69 ) | 0.62 ( 71.91 ) | 0.62 ( 0.57 ) | -0.69 ( -0.85 ) |
| Cough | 294 | 0.99 ( 0.88 - 1.11 ) | 0.99 ( 0.02 ) | 0.99 ( 0.9 ) | -0.01 ( -0.18 ) |
| Constipation* | 284 | 1.39 ( 1.24 - 1.56 ) | 1.39 ( 30.95 ) | 1.39 ( 1.26 ) | 0.47 ( 0.3 ) |
| Sepsis* | 282 | 3.62 ( 3.22 - 4.07 ) | 3.6 ( 526.54 ) | 3.58 ( 3.25 ) | 1.84 ( 1.67 ) |
| Abdominal pain* | 252 | 1.15 ( 1.02 - 1.3 ) | 1.15 ( 4.92 ) | 1.15 ( 1.04 ) | 0.2 ( 0.02 ) |
| Pleural effusion* | 247 | 5.46 ( 4.81 - 6.19 ) | 5.44 ( 882.79 ) | 5.38 ( 4.84 ) | 2.43 ( 2.24 ) |
| Urinary tract infection | 228 | 1.11 ( 0.97 - 1.26 ) | 1.1 ( 2.27 ) | 1.1 ( 0.99 ) | 0.14 ( -0.05 ) |
| Immune-mediated enterocolitis* | 227 | 149.02 ( 127.81 - 173.74 ) | 148.43 ( 23948.89 ) | 107.21 ( 94.29 ) | 6.74 ( 6.53 ) |
| Dizziness | 220 | 0.44 ( 0.39 - 0.5 ) | 0.44 ( 154.53 ) | 0.44 ( 0.4 ) | -1.17 ( -1.36 ) |
| Hypotension* | 215 | 1.33 ( 1.16 - 1.52 ) | 1.33 ( 17.61 ) | 1.33 ( 1.19 ) | 0.41 ( 0.21 ) |
| Dehydration* | 215 | 1.96 ( 1.71 - 2.24 ) | 1.96 ( 100.28 ) | 1.95 ( 1.74 ) | 0.96 ( 0.77 ) |
| Fall | 214 | 0.64 ( 0.56 - 0.73 ) | 0.64 ( 42.95 ) | 0.64 ( 0.57 ) | -0.64 ( -0.84 ) |
| Thrombocytopenia* | 212 | 2.98 ( 2.6 - 3.41 ) | 2.97 ( 275.43 ) | 2.96 ( 2.64 ) | 1.56 ( 1.36 ) |
| Drug ineffective | 207 | 0.15 ( 0.13 - 0.18 ) | 0.16 ( 955.19 ) | 0.16 ( 0.14 ) | -2.66 ( -2.86 ) |
| Product use issue | 199 | 0.86 ( 0.75 - 0.99 ) | 0.86 ( 4.55 ) | 0.86 ( 0.77 ) | -0.22 ( -0.42 ) |
| Respiratory failure* | 195 | 4.17 ( 3.62 - 4.8 ) | 4.16 ( 462.72 ) | 4.12 ( 3.66 ) | 2.04 ( 1.84 ) |
| Hyperthyroidism* | 193 | 14.69 ( 12.72 - 16.97 ) | 14.65 ( 2364.25 ) | 14.14 ( 12.54 ) | 3.82 ( 3.61 ) |
| Hepatic function abnormal* | 193 | 8.45 ( 7.32 - 9.75 ) | 8.42 ( 1235.8 ) | 8.26 ( 7.33 ) | 3.05 ( 2.84 ) |
| Adrenal insufficiency* | 191 | 21.81 ( 18.84 - 25.24 ) | 21.74 ( 3576.05 ) | 20.62 ( 18.25 ) | 4.37 ( 4.15 ) |
| Myocarditis* | 189 | 29.1 ( 25.1 - 33.75 ) | 29.01 ( 4751.78 ) | 27.04 ( 23.88 ) | 4.76 ( 4.54 ) |
| Interstitial lung disease* | 181 | 5.64 ( 4.87 - 6.53 ) | 5.63 ( 678.82 ) | 5.56 ( 4.92 ) | 2.47 ( 2.26 ) |
| Hepatitis* | 175 | 9.34 ( 8.04 - 10.86 ) | 9.32 ( 1268.94 ) | 9.12 ( 8.04 ) | 3.19 ( 2.97 ) |

Abbreviation: Asterisks (*) indicate statistically significant signals in algorithm; ROR, reporting odds ratio; PRR, proportional reporting ratio; EBGM, empirical Bayesian geometric mean; EBGM05, the lower limit of the 95% CI of EBGM; IC, information component; IC025, the lower limit of the 95% CI of the IC; CI, confidence interval; PT,preferred term; AEs, adverse events.

Supplementary Table 6:

Top 50 most frequent adverse events at the PT level for Nivolumab in patients aged under 18 from FAERS data

| PT | Case numbers | ROR(95%CI) | PRR(χ^2^) | EBGM(EBGM05) | IC(IC025) |
| --- | --- | --- | --- | --- | --- |
| Malignant neoplasm progression* | 41 | 47.55 ( 34.62 - 65.31 ) | 45.83 ( 1735.81 ) | 44.25 ( 33.93 ) | 5.47 ( 5.01 ) |
| Intentional product use issue* | 41 | 29.87 ( 21.79 - 40.94 ) | 28.8 ( 1077.03 ) | 28.18 ( 21.64 ) | 4.82 ( 4.36 ) |
| Off label use | 34 | 0.95 ( 0.68 - 1.34 ) | 0.95 ( 0.08 ) | 0.95 ( 0.72 ) | -0.07 ( -0.57 ) |
| Pyrexia* | 26 | 1.96 ( 1.32 - 2.89 ) | 1.93 ( 11.83 ) | 1.93 ( 1.39 ) | 0.95 ( 0.39 ) |
| Death* | 23 | 5.89 ( 3.89 - 8.91 ) | 5.79 ( 91 ) | 5.77 ( 4.08 ) | 2.53 ( 1.93 ) |
| Cytokine release syndrome* | 16 | 29.35 ( 17.82 - 48.36 ) | 28.95 ( 422.14 ) | 28.31 ( 18.65 ) | 4.82 ( 4.11 ) |
| Diarrhoea* | 14 | 1.76 ( 1.04 - 2.98 ) | 1.75 ( 4.54 ) | 1.75 ( 1.13 ) | 0.81 ( 0.06 ) |
| Febrile neutropenia* | 14 | 3.23 ( 1.91 - 5.48 ) | 3.2 ( 21.23 ) | 3.2 ( 2.06 ) | 1.68 ( 0.93 ) |
| Pneumonia* | 13 | 2.82 ( 1.63 - 4.87 ) | 2.8 ( 15.05 ) | 2.79 ( 1.77 ) | 1.48 ( 0.71 ) |
| Rash | 12 | 1.13 ( 0.64 - 1.99 ) | 1.13 ( 0.17 ) | 1.13 ( 0.7 ) | 0.17 ( -0.63 ) |
| Vomiting | 11 | 0.82 ( 0.45 - 1.48 ) | 0.82 ( 0.45 ) | 0.82 ( 0.5 ) | -0.29 ( -1.13 ) |
| Seizure | 10 | 1.18 ( 0.63 - 2.2 ) | 1.18 ( 0.27 ) | 1.18 ( 0.7 ) | 0.24 ( -0.64 ) |
| Dehydration* | 8 | 4.74 ( 2.36 - 9.51 ) | 4.71 ( 23.35 ) | 4.7 ( 2.62 ) | 2.23 ( 1.26 ) |
| Dyspnoea | 8 | 1.56 ( 0.78 - 3.12 ) | 1.55 ( 1.57 ) | 1.55 ( 0.87 ) | 0.63 ( -0.33 ) |
| Platelet count decreased* | 8 | 5.27 ( 2.62 - 10.57 ) | 5.24 ( 27.33 ) | 5.22 ( 2.91 ) | 2.38 ( 1.42 ) |
| Immune-mediated enterocolitis* | 8 | 305.69 ( 140.88 - 663.29 ) | 303.49 ( 1941.37 ) | 244.47 ( 127.85 ) | 7.93 ( 6.87 ) |
| Adrenal insufficiency* | 8 | 17 ( 8.44 - 34.25 ) | 16.89 ( 118.04 ) | 16.68 ( 9.28 ) | 4.06 ( 3.09 ) |
| Product use issue | 7 | 0.56 ( 0.26 - 1.17 ) | 0.56 ( 2.45 ) | 0.56 ( 0.3 ) | -0.84 ( -1.86 ) |
| Sepsis* | 7 | 3.55 ( 1.69 - 7.47 ) | 3.53 ( 12.7 ) | 3.53 ( 1.89 ) | 1.82 ( 0.79 ) |
| Product use in unapproved indication | 7 | 0.64 ( 0.3 - 1.34 ) | 0.64 ( 1.43 ) | 0.64 ( 0.34 ) | -0.64 ( -1.67 ) |
| Nausea | 7 | 0.9 ( 0.43 - 1.89 ) | 0.9 ( 0.08 ) | 0.9 ( 0.48 ) | -0.15 ( -1.18 ) |
| Decreased appetite | 7 | 1.96 ( 0.93 - 4.13 ) | 1.96 ( 3.28 ) | 1.96 ( 1.05 ) | 0.97 ( -0.06 ) |
| Fulminant type 1 diabetes mellitus* | 7 | 881.88 ( 335.08 - 2321.01 ) | 876.32 ( 3600.21 ) | 515.9 ( 229.57 ) | 9.01 ( 7.78 ) |
| Immune-mediated hepatitis* | 7 | 629.91 ( 253.75 - 1563.69 ) | 625.95 ( 2911.8 ) | 417.63 ( 195.16 ) | 8.71 ( 7.51 ) |
| Pneumonia aspiration* | 6 | 11.76 ( 5.25 - 26.33 ) | 11.7 ( 58.2 ) | 11.6 ( 5.91 ) | 3.54 ( 2.44 ) |
| Interstitial lung disease* | 6 | 13.53 ( 6.04 - 30.31 ) | 13.46 ( 68.5 ) | 13.33 ( 6.79 ) | 3.74 ( 2.64 ) |
| Skin disorder* | 6 | 12.64 ( 5.65 - 28.32 ) | 12.58 ( 63.36 ) | 12.47 ( 6.35 ) | 3.64 ( 2.54 ) |
| Immune-mediated cholangitis* | 6 | 1510.43 ( 460.28 - 4956.5 ) | 1502.27 ( 4091.68 ) | 683.4 ( 252.85 ) | 9.42 ( 8.04 ) |
| Hepatic function abnormal* | 6 | 6.98 ( 3.12 - 15.61 ) | 6.95 ( 30.41 ) | 6.92 ( 3.53 ) | 2.79 ( 1.69 ) |
| Pain | 5 | 1.18 ( 0.49 - 2.83 ) | 1.18 ( 0.13 ) | 1.18 ( 0.56 ) | 0.23 ( -0.95 ) |
| Hydrocephalus* | 5 | 13.29 ( 5.49 - 32.14 ) | 13.23 ( 55.97 ) | 13.11 ( 6.26 ) | 3.71 ( 2.52 ) |
| Alanine aminotransferase increased* | 5 | 2.87 ( 1.19 - 6.91 ) | 2.86 ( 6.05 ) | 2.86 ( 1.37 ) | 1.51 ( 0.33 ) |
| Anaemia | 5 | 2.25 ( 0.93 - 5.41 ) | 2.24 ( 3.43 ) | 2.24 ( 1.07 ) | 1.16 ( -0.02 ) |
| Hypercalcaemia* | 5 | 21.24 ( 8.76 - 51.5 ) | 21.15 ( 94.4 ) | 20.81 ( 9.92 ) | 4.38 ( 3.19 ) |
| Abdominal pain | 5 | 0.9 ( 0.37 - 2.16 ) | 0.9 ( 0.06 ) | 0.9 ( 0.43 ) | -0.15 ( -1.33 ) |
| Ascites* | 5 | 9.24 ( 3.83 - 22.32 ) | 9.21 ( 36.32 ) | 9.15 ( 4.37 ) | 3.19 ( 2.01 ) |
| Cough | 5 | 1 ( 0.41 - 2.4 ) | 1 ( 0 ) | 1 ( 0.48 ) | 0 ( -1.19 ) |
| Gastritis* | 5 | 14.45 ( 5.97 - 34.96 ) | 14.39 ( 61.61 ) | 14.24 ( 6.8 ) | 3.83 ( 2.64 ) |
| Malaise | 5 | 1.16 ( 0.48 - 2.79 ) | 1.16 ( 0.11 ) | 1.16 ( 0.55 ) | 0.21 ( -0.97 ) |
| Immune-mediated hypothyroidism* | 5 | 314.38 ( 117.78 - 839.15 ) | 312.97 ( 1243.93 ) | 250.58 ( 110.2 ) | 7.97 ( 6.66 ) |
| Colitis* | 5 | 7.32 ( 3.03 - 17.68 ) | 7.3 ( 27.02 ) | 7.26 ( 3.47 ) | 2.86 ( 1.67 ) |
| Diabetic ketoacidosis* | 5 | 11.3 ( 4.68 - 27.32 ) | 11.26 ( 46.34 ) | 11.17 ( 5.34 ) | 3.48 ( 2.29 ) |
| Proteinuria* | 5 | 9.23 ( 3.82 - 22.29 ) | 9.19 ( 36.25 ) | 9.13 ( 4.37 ) | 3.19 ( 2 ) |
| Myelosuppression* | 5 | 4.59 ( 1.9 - 11.06 ) | 4.57 ( 13.9 ) | 4.56 ( 2.18 ) | 2.19 ( 1 ) |
| Headache* | 4 | 0.44 ( 0.17 - 1.18 ) | 0.44 ( 2.79 ) | 0.44 ( 0.2 ) | -1.17 ( -2.46 ) |
| Hypertension | 4 | 1.65 ( 0.62 - 4.41 ) | 1.65 ( 1.02 ) | 1.65 ( 0.72 ) | 0.72 ( -0.57 ) |
| Aspartate aminotransferase increased* | 4 | 2.69 ( 1.01 - 7.2 ) | 2.69 ( 4.24 ) | 2.68 ( 1.18 ) | 1.42 ( 0.13 ) |
| Hypoxia* | 4 | 3.3 ( 1.23 - 8.81 ) | 3.29 ( 6.36 ) | 3.28 ( 1.44 ) | 1.71 ( 0.42 ) |
| Pleural effusion* | 4 | 4.33 ( 1.62 - 11.57 ) | 4.32 ( 10.17 ) | 4.31 ( 1.89 ) | 2.11 ( 0.81 ) |
| Liver transplant rejection* | 4 | 40.2 ( 14.83 - 108.99 ) | 40.06 ( 147.63 ) | 38.85 ( 16.86 ) | 5.28 ( 3.96 ) |

Abbreviation: Asterisks (*) indicate statistically significant signals in algorithm; ROR, reporting odds ratio; PRR, proportional reporting ratio; EBGM, empirical Bayesian geometric mean; EBGM05, the lower limit of the 95% CI of EBGM; IC, information component; IC025, the lower limit of the 95% CI of the IC; CI, confidence interval; PT, preferred term.

Supplementary Table 7:

Top 50 most frequent adverse events for Nivolumab at the PT level in patients aged 18 to 65 from FAERS data

| PT | Case numbers | ROR(95%CI) | PRR(χ^2^) | EBGM(EBGM05) | IC(IC025) |
| --- | --- | --- | --- | --- | --- |
| Malignant neoplasm progression* | 2718 | 35.49 ( 34.06 - 36.98 ) | 33.93 ( 76073.34 ) | 29.79 ( 28.79 ) | 4.9 ( 4.84 ) |
| Death* | 2606 | 6.18 ( 5.94 - 6.43 ) | 5.96 ( 10563.91 ) | 5.84 ( 5.64 ) | 2.54 ( 2.49 ) |
| Off label use* | 1421 | 1.6 ( 1.52 - 1.68 ) | 1.58 ( 308.5 ) | 1.58 ( 1.51 ) | 0.66 ( 0.58 ) |
| Diarrhoea* | 1068 | 1.73 ( 1.63 - 1.84 ) | 1.71 ( 318.76 ) | 1.71 ( 1.62 ) | 0.77 ( 0.68 ) |
| Intentional product use issue* | 986 | 10.18 ( 9.54 - 10.85 ) | 10.03 ( 7699.43 ) | 9.66 ( 9.15 ) | 3.27 ( 3.18 ) |
| Pyrexia* | 905 | 2.4 ( 2.25 - 2.57 ) | 2.38 ( 722.2 ) | 2.37 ( 2.24 ) | 1.24 ( 1.15 ) |
| Fatigue | 898 | 1.06 ( 0.99 - 1.13 ) | 1.06 ( 3.12 ) | 1.06 ( 1 ) | 0.08 ( -0.01 ) |
| Nausea | 735 | 0.84 ( 0.78 - 0.9 ) | 0.84 ( 21.83 ) | 0.84 ( 0.79 ) | -0.25 ( -0.35 ) |
| Dyspnoea* | 673 | 1.26 ( 1.17 - 1.36 ) | 1.25 ( 34.85 ) | 1.25 ( 1.18 ) | 0.33 ( 0.21 ) |
| Vomiting* | 570 | 1.18 ( 1.08 - 1.28 ) | 1.18 ( 14.99 ) | 1.17 ( 1.1 ) | 0.23 ( 0.11 ) |
| Rash* | 540 | 1.28 ( 1.18 - 1.4 ) | 1.28 ( 33.57 ) | 1.28 ( 1.19 ) | 0.36 ( 0.23 ) |
| Decreased appetite* | 531 | 2.67 ( 2.45 - 2.91 ) | 2.65 ( 542.13 ) | 2.63 ( 2.45 ) | 1.4 ( 1.27 ) |
| Pneumonia* | 518 | 1.91 ( 1.75 - 2.08 ) | 1.9 ( 220.65 ) | 1.89 ( 1.76 ) | 0.92 ( 0.79 ) |
| Colitis* | 512 | 13.86 ( 12.68 - 15.16 ) | 13.75 ( 5725.09 ) | 13.05 ( 12.11 ) | 3.71 ( 3.57 ) |
| Hypothyroidism* | 453 | 15.79 ( 14.35 - 17.37 ) | 15.68 ( 5840.86 ) | 14.77 ( 13.63 ) | 3.88 ( 3.74 ) |
| Asthenia* | 448 | 1.35 ( 1.23 - 1.48 ) | 1.35 ( 39.87 ) | 1.34 ( 1.24 ) | 0.43 ( 0.29 ) |
| Pneumonitis* | 438 | 19.46 ( 17.65 - 21.46 ) | 19.32 ( 7037.61 ) | 17.94 ( 16.53 ) | 4.16 ( 4.02 ) |
| Anaemia* | 435 | 3.01 ( 2.74 - 3.31 ) | 2.99 ( 571.38 ) | 2.97 ( 2.74 ) | 1.57 ( 1.43 ) |
| Headache | 422 | 0.54 ( 0.49 - 0.59 ) | 0.54 ( 164.85 ) | 0.54 ( 0.5 ) | -0.88 ( -1.02 ) |
| Weight decreased* | 404 | 1.66 ( 1.51 - 1.83 ) | 1.66 ( 105.09 ) | 1.65 ( 1.52 ) | 0.73 ( 0.58 ) |
| Acute kidney injury* | 391 | 2.16 ( 1.95 - 2.39 ) | 2.15 ( 239.52 ) | 2.14 ( 1.97 ) | 1.1 ( 0.95 ) |
| General physical health deterioration* | 376 | 4.37 ( 3.95 - 4.84 ) | 4.35 ( 953.72 ) | 4.29 ( 3.94 ) | 2.1 ( 1.95 ) |
| Arthralgia | 349 | 0.72 ( 0.65 - 0.8 ) | 0.72 ( 37.15 ) | 0.72 ( 0.66 ) | -0.47 ( -0.62 ) |
| Pain | 337 | 0.53 ( 0.48 - 0.59 ) | 0.54 ( 135.75 ) | 0.54 ( 0.49 ) | -0.89 ( -1.05 ) |
| Pruritus | 325 | 0.83 ( 0.74 - 0.92 ) | 0.83 ( 11.32 ) | 0.83 ( 0.76 ) | -0.27 ( -0.43 ) |
| Back pain* | 317 | 1.3 ( 1.17 - 1.45 ) | 1.3 ( 21.92 ) | 1.3 ( 1.18 ) | 0.38 ( 0.21 ) |
| Cough | 298 | 1.07 ( 0.95 - 1.19 ) | 1.07 ( 1.19 ) | 1.06 ( 0.97 ) | 0.09 ( -0.08 ) |
| Abdominal pain | 294 | 1.09 ( 0.97 - 1.22 ) | 1.09 ( 2.21 ) | 1.09 ( 0.99 ) | 0.12 ( -0.04 ) |
| Sepsis* | 281 | 2.91 ( 2.59 - 3.27 ) | 2.9 ( 346.28 ) | 2.88 ( 2.61 ) | 1.52 ( 1.35 ) |
| Pleural effusion* | 274 | 5.69 ( 5.04 - 6.41 ) | 5.66 ( 1028.7 ) | 5.56 ( 5.02 ) | 2.47 ( 2.3 ) |
| Constipation* | 271 | 1.61 ( 1.43 - 1.82 ) | 1.61 ( 62.29 ) | 1.61 ( 1.45 ) | 0.68 ( 0.51 ) |
| Immune-mediated enterocolitis* | 266 | 111.73 ( 96.56 - 129.29 ) | 111.24 ( 19759.68 ) | 75.95 ( 67.22 ) | 6.25 ( 6.04 ) |
| Malaise | 259 | 0.6 ( 0.53 - 0.67 ) | 0.6 ( 69.83 ) | 0.6 ( 0.54 ) | -0.74 ( -0.92 ) |
| Thrombocytopenia* | 252 | 2.56 ( 2.26 - 2.9 ) | 2.55 ( 235.93 ) | 2.54 ( 2.29 ) | 1.34 ( 1.16 ) |
| Adrenal insufficiency* | 244 | 22 ( 19.29 - 25.09 ) | 21.91 ( 4457.53 ) | 20.14 ( 18.04 ) | 4.33 ( 4.14 ) |
| Hyperthyroidism* | 241 | 16.16 ( 14.18 - 18.42 ) | 16.1 ( 3196.13 ) | 15.14 ( 13.57 ) | 3.92 ( 3.73 ) |
| Interstitial lung disease* | 241 | 7.41 ( 6.51 - 8.42 ) | 7.38 ( 1289.93 ) | 7.19 ( 6.46 ) | 2.85 ( 2.66 ) |
| Hypotension* | 238 | 1.29 ( 1.14 - 1.47 ) | 1.29 ( 15.78 ) | 1.29 ( 1.16 ) | 0.37 ( 0.18 ) |
| Hepatic function abnormal* | 211 | 6.04 ( 5.27 - 6.93 ) | 6.02 ( 862.8 ) | 5.9 ( 5.26 ) | 2.56 ( 2.36 ) |
| Dehydration* | 210 | 2.09 ( 1.83 - 2.4 ) | 2.09 ( 118.4 ) | 2.08 ( 1.86 ) | 1.06 ( 0.86 ) |
| Respiratory failure* | 208 | 3.34 ( 2.91 - 3.83 ) | 3.33 ( 335.16 ) | 3.3 ( 2.94 ) | 1.72 ( 1.52 ) |
| Alanine aminotransferase increased* | 206 | 3.4 ( 2.96 - 3.9 ) | 3.39 ( 342.78 ) | 3.36 ( 2.99 ) | 1.75 ( 1.55 ) |
| Aspartate aminotransferase increased* | 206 | 4.32 ( 3.76 - 4.96 ) | 4.31 ( 514.79 ) | 4.25 ( 3.79 ) | 2.09 ( 1.89 ) |
| Dizziness | 204 | 0.42 ( 0.36 - 0.48 ) | 0.42 ( 167.12 ) | 0.42 ( 0.37 ) | -1.26 ( -1.46 ) |
| Product use in unapproved indication | 194 | 0.71 ( 0.62 - 0.82 ) | 0.71 ( 22.38 ) | 0.71 ( 0.63 ) | -0.49 ( -0.69 ) |
| Pulmonary embolism* | 191 | 2.39 ( 2.07 - 2.76 ) | 2.38 ( 152.26 ) | 2.37 ( 2.1 ) | 1.25 ( 1.04 ) |
| Prescribed underdose* | 188 | 8.72 ( 7.54 - 10.09 ) | 8.7 ( 1236.15 ) | 8.43 ( 7.46 ) | 3.07 ( 2.86 ) |
| Febrile neutropenia* | 185 | 3.02 ( 2.62 - 3.5 ) | 3.02 ( 246.67 ) | 2.99 ( 2.65 ) | 1.58 ( 1.37 ) |
| Diabetic ketoacidosis* | 184 | 5.25 ( 4.54 - 6.08 ) | 5.24 ( 618.06 ) | 5.15 ( 4.56 ) | 2.36 ( 2.15 ) |
| Hypophysitis* | 183 | 96.59 ( 81.32 - 114.73 ) | 96.3 ( 12262.35 ) | 68.71 ( 59.49 ) | 6.1 ( 5.86 ) |

Abbreviation: Asterisks (*) indicate statistically significant signals in algorithm; ROR, reporting odds ratio; PRR, proportional reporting ratio; EBGM, empirical Bayesian geometric mean; EBGM05, the lower limit of the 95% CI of EBGM; IC, information component; IC025, the lower limit of the 95% CI of the IC; CI, confidence interval; PT, preferred term.

Supplementary Table 8:

Top 50 most frequent adverse events for Nivolumab at the PT level in patients aged 65 to 85 from FAERS data

| PT | Case numbers | ROR(95%CI) | PRR(χ^2^) | EBGM(EBGM05) | IC(IC025) |
| --- | --- | --- | --- | --- | --- |
| Malignant neoplasm progression* | 2889 | 19.62 ( 18.85 - 20.42 ) | 18.85 ( 42042.66 ) | 16.33 ( 15.79 ) | 4.03 ( 3.97 ) |
| Death* | 2497 | 1.9 ( 1.82 - 1.97 ) | 1.86 ( 1004.01 ) | 1.85 ( 1.79 ) | 0.89 ( 0.83 ) |
| Off label use* | 1353 | 1.19 ( 1.13 - 1.26 ) | 1.19 ( 41.59 ) | 1.19 ( 1.14 ) | 0.25 ( 0.17 ) |
| Diarrhoea* | 1277 | 1.3 ( 1.23 - 1.37 ) | 1.29 ( 84.39 ) | 1.29 ( 1.23 ) | 0.37 ( 0.28 ) |
| Fatigue | 994 | 1.02 ( 0.95 - 1.08 ) | 1.01 ( 0.22 ) | 1.01 ( 0.96 ) | 0.02 ( -0.07 ) |
| Intentional product use issue* | 938 | 8.58 ( 8.03 - 9.18 ) | 8.48 ( 5774.13 ) | 7.97 ( 7.53 ) | 2.99 ( 2.9 ) |
| Pyrexia* | 878 | 2.25 ( 2.1 - 2.4 ) | 2.23 ( 588.48 ) | 2.21 ( 2.09 ) | 1.14 ( 1.04 ) |
| Decreased appetite* | 856 | 2.14 ( 2 - 2.29 ) | 2.13 ( 506.58 ) | 2.11 ( 1.99 ) | 1.08 ( 0.98 ) |
| Dyspnoea | 839 | 0.99 ( 0.93 - 1.06 ) | 0.99 ( 0.06 ) | 0.99 ( 0.94 ) | -0.01 ( -0.11 ) |
| Pneumonia* | 794 | 1.42 ( 1.33 - 1.53 ) | 1.42 ( 97.19 ) | 1.41 ( 1.33 ) | 0.5 ( 0.39 ) |
| Asthenia* | 683 | 1.14 ( 1.05 - 1.23 ) | 1.14 ( 11.1 ) | 1.13 ( 1.06 ) | 0.18 ( 0.07 ) |
| Nausea | 657 | 0.77 ( 0.71 - 0.83 ) | 0.77 ( 43.52 ) | 0.78 ( 0.73 ) | -0.37 ( -0.48 ) |
| Rash* | 604 | 1.34 ( 1.24 - 1.45 ) | 1.34 ( 51.07 ) | 1.33 ( 1.25 ) | 0.41 ( 0.3 ) |
| Acute kidney injury* | 597 | 1.64 ( 1.51 - 1.78 ) | 1.64 ( 146.09 ) | 1.63 ( 1.52 ) | 0.7 ( 0.58 ) |
| General physical health deterioration* | 556 | 3.08 ( 2.83 - 3.35 ) | 3.06 ( 754.86 ) | 3.01 ( 2.8 ) | 1.59 ( 1.47 ) |
| Interstitial lung disease* | 545 | 5.1 ( 4.68 - 5.55 ) | 5.06 ( 1704.97 ) | 4.89 ( 4.55 ) | 2.29 ( 2.16 ) |
| Hypothyroidism* | 526 | 13.97 ( 12.76 - 15.3 ) | 13.87 ( 5608.87 ) | 12.48 ( 11.57 ) | 3.64 ( 3.51 ) |
| Weight decreased* | 510 | 1.33 ( 1.22 - 1.45 ) | 1.33 ( 40.84 ) | 1.32 ( 1.23 ) | 0.4 ( 0.28 ) |
| Pneumonitis* | 502 | 10.92 ( 9.97 - 11.98 ) | 10.85 ( 4105.41 ) | 10 ( 9.26 ) | 3.32 ( 3.19 ) |
| Colitis* | 498 | 10.07 ( 9.19 - 11.04 ) | 10 ( 3715.44 ) | 9.28 ( 8.6 ) | 3.21 ( 3.08 ) |
| Anaemia* | 496 | 1.47 ( 1.35 - 1.61 ) | 1.47 ( 73.21 ) | 1.46 ( 1.36 ) | 0.55 ( 0.42 ) |
| Vomiting | 469 | 0.97 ( 0.88 - 1.06 ) | 0.97 ( 0.58 ) | 0.97 ( 0.89 ) | -0.05 ( -0.18 ) |
| Pruritus* | 454 | 1.14 ( 1.04 - 1.25 ) | 1.14 ( 7.58 ) | 1.14 ( 1.05 ) | 0.18 ( 0.05 ) |
| Malaise | 395 | 0.7 ( 0.63 - 0.77 ) | 0.7 ( 50.78 ) | 0.7 ( 0.65 ) | -0.51 ( -0.66 ) |
| Sepsis* | 386 | 2.24 ( 2.03 - 2.48 ) | 2.24 ( 259.75 ) | 2.21 ( 2.03 ) | 1.15 ( 1 ) |
| Arthralgia | 379 | 0.79 ( 0.72 - 0.88 ) | 0.79 ( 20.05 ) | 0.8 ( 0.73 ) | -0.33 ( -0.48 ) |
| Constipation* | 371 | 1.14 ( 1.03 - 1.26 ) | 1.14 ( 5.98 ) | 1.13 ( 1.04 ) | 0.18 ( 0.03 ) |
| Myocarditis* | 358 | 33.6 ( 29.86 - 37.8 ) | 33.43 ( 8723.06 ) | 26.11 ( 23.66 ) | 4.71 ( 4.54 ) |
| Dehydration* | 351 | 1.71 ( 1.54 - 1.9 ) | 1.7 ( 100.9 ) | 1.69 ( 1.55 ) | 0.76 ( 0.6 ) |
| Adrenal insufficiency* | 347 | 23.13 ( 20.6 - 25.96 ) | 23.02 ( 6088.76 ) | 19.34 ( 17.56 ) | 4.27 ( 4.11 ) |
| Fall | 338 | 0.51 ( 0.46 - 0.57 ) | 0.52 ( 153.12 ) | 0.52 ( 0.47 ) | -0.95 ( -1.1 ) |
| Cough | 329 | 0.82 ( 0.73 - 0.91 ) | 0.82 ( 13.65 ) | 0.82 ( 0.75 ) | -0.29 ( -0.45 ) |
| Pleural effusion* | 318 | 3.07 ( 2.74 - 3.43 ) | 3.06 ( 429.19 ) | 3 ( 2.74 ) | 1.59 ( 1.42 ) |
| Hepatic function abnormal* | 300 | 5.99 ( 5.33 - 6.73 ) | 5.97 ( 1180.38 ) | 5.72 ( 5.19 ) | 2.52 ( 2.35 ) |
| Pemphigoid* | 300 | 16.36 ( 14.49 - 18.47 ) | 16.29 ( 3772.01 ) | 14.39 ( 13 ) | 3.85 ( 3.67 ) |
| Respiratory failure* | 292 | 2.6 ( 2.31 - 2.92 ) | 2.59 ( 279.03 ) | 2.55 ( 2.32 ) | 1.35 ( 1.18 ) |
| Pain | 292 | 0.52 ( 0.46 - 0.58 ) | 0.52 ( 130.12 ) | 0.52 ( 0.47 ) | -0.94 ( -1.11 ) |
| Atrial fibrillation* | 290 | 1.28 ( 1.14 - 1.44 ) | 1.28 ( 17.7 ) | 1.28 ( 1.16 ) | 0.35 ( 0.18 ) |
| Back pain | 288 | 0.92 ( 0.82 - 1.04 ) | 0.92 ( 1.87 ) | 0.92 ( 0.84 ) | -0.12 ( -0.29 ) |
| Immune-mediated enterocolitis* | 288 | 54.13 ( 47.05 - 62.28 ) | 53.91 ( 10177.05 ) | 37 ( 32.9 ) | 5.21 ( 5.01 ) |
| Cardiac failure* | 270 | 1.79 ( 1.58 - 2.01 ) | 1.78 ( 91.67 ) | 1.77 ( 1.6 ) | 0.82 ( 0.65 ) |
| Hypotension | 267 | 0.84 ( 0.74 - 0.95 ) | 0.84 ( 8.25 ) | 0.84 ( 0.76 ) | -0.25 ( -0.43 ) |
| Renal failure* | 267 | 1.66 ( 1.47 - 1.88 ) | 1.66 ( 69.21 ) | 1.65 ( 1.49 ) | 0.72 ( 0.55 ) |
| Thrombocytopenia* | 262 | 1.54 ( 1.36 - 1.74 ) | 1.54 ( 49.03 ) | 1.53 ( 1.38 ) | 0.62 ( 0.44 ) |
| Prescribed underdose* | 255 | 7.85 ( 6.91 - 8.91 ) | 7.82 ( 1421.14 ) | 7.39 ( 6.64 ) | 2.88 ( 2.7 ) |
| Dizziness | 233 | 0.36 ( 0.32 - 0.41 ) | 0.36 ( 264.2 ) | 0.36 ( 0.33 ) | -1.46 ( -1.65 ) |
| Myositis* | 231 | 21.04 ( 18.28 - 24.21 ) | 20.97 ( 3715.37 ) | 17.89 ( 15.9 ) | 4.16 ( 3.96 ) |
| Headache | 231 | 0.41 ( 0.36 - 0.47 ) | 0.41 ( 195.94 ) | 0.41 ( 0.37 ) | -1.28 ( -1.47 ) |
| Hyponatraemia* | 227 | 1.91 ( 1.68 - 2.18 ) | 1.91 ( 96.67 ) | 1.89 ( 1.7 ) | 0.92 ( 0.73 ) |
| Lung disorder* | 227 | 3.1 ( 2.71 - 3.53 ) | 3.09 ( 312.98 ) | 3.04 ( 2.72 ) | 1.6 ( 1.41 ) |

Abbreviation: Asterisks (*) indicate statistically significant signals in algorithm; ROR, reporting odds ratio; PRR, proportional reporting ratio; EBGM, empirical Bayesian geometric mean; EBGM05, the lower limit of the 95% CI of EBGM; IC, information component; IC025, the lower limit of the 95% CI of the IC; CI, confidence interval; PT, preferred term.

Supplementary Table 9:

Top 50 most frequent adverse events for Nivolumab at the PT level in patients over 85 from FAERS data

| PT | Case numbers | ROR(95%CI) | PRR(χ^2^) | EBGM(EBGM05) | IC(IC025) |
| --- | --- | --- | --- | --- | --- |
| Death* | 154 | 1.6 ( 1.36 - 1.89 ) | 1.55 ( 31.87 ) | 1.55 ( 1.35 ) | 0.63 ( 0.39 ) |
| Malignant neoplasm progression* | 72 | 26.55 ( 20.81 - 33.88 ) | 25.61 ( 1586.51 ) | 23.89 ( 19.49 ) | 4.58 ( 4.22 ) |
| Off label use* | 67 | 1.82 ( 1.42 - 2.32 ) | 1.79 ( 23.74 ) | 1.79 ( 1.46 ) | 0.84 ( 0.48 ) |
| Intentional product use issue* | 53 | 14.18 ( 10.74 - 18.73 ) | 13.82 ( 607.32 ) | 13.33 ( 10.56 ) | 3.74 ( 3.33 ) |
| Diarrhoea* | 37 | 1.6 ( 1.16 - 2.22 ) | 1.59 ( 8.22 ) | 1.59 ( 1.21 ) | 0.67 ( 0.2 ) |
| Fatigue | 30 | 1.36 ( 0.95 - 1.95 ) | 1.35 ( 2.81 ) | 1.35 ( 1 ) | 0.44 ( -0.09 ) |
| Asthenia* | 30 | 1.49 ( 1.04 - 2.14 ) | 1.48 ( 4.7 ) | 1.48 ( 1.09 ) | 0.56 ( 0.04 ) |
| Dyspnoea | 26 | 1.15 ( 0.78 - 1.7 ) | 1.15 ( 0.53 ) | 1.15 ( 0.83 ) | 0.2 ( -0.36 ) |
| Pneumonia | 22 | 1.27 ( 0.83 - 1.93 ) | 1.27 ( 1.23 ) | 1.26 ( 0.89 ) | 0.34 ( -0.27 ) |
| Interstitial lung disease* | 21 | 9.38 ( 6.07 - 14.51 ) | 9.29 ( 151.47 ) | 9.07 ( 6.3 ) | 3.18 ( 2.55 ) |
| Decreased appetite | 18 | 1.51 ( 0.95 - 2.41 ) | 1.51 ( 3.09 ) | 1.51 ( 1.02 ) | 0.59 ( -0.08 ) |
| Pyrexia* | 17 | 2.34 ( 1.45 - 3.77 ) | 2.32 ( 12.79 ) | 2.32 ( 1.55 ) | 1.21 ( 0.53 ) |
| Acute kidney injury | 17 | 1 ( 0.62 - 1.61 ) | 1 ( 0 ) | 1 ( 0.67 ) | 0 ( -0.69 ) |
| Pruritus* | 17 | 1.7 ( 1.05 - 2.74 ) | 1.69 ( 4.82 ) | 1.69 ( 1.13 ) | 0.76 ( 0.07 ) |
| Vomiting | 16 | 1.42 ( 0.86 - 2.32 ) | 1.41 ( 1.93 ) | 1.41 ( 0.93 ) | 0.5 ( -0.21 ) |
| Hypothyroidism* | 16 | 19.58 ( 11.81 - 32.47 ) | 19.43 ( 264.83 ) | 18.44 ( 12.08 ) | 4.2 ( 3.48 ) |
| Cardiac failure* | 16 | 1.81 ( 1.1 - 2.96 ) | 1.8 ( 5.67 ) | 1.79 ( 1.19 ) | 0.84 ( 0.14 ) |
| Myocarditis* | 16 | 110.49 ( 62.82 - 194.36 ) | 109.6 ( 1304.44 ) | 83.27 ( 51.91 ) | 6.38 ( 5.59 ) |
| Rash | 15 | 1.49 ( 0.9 - 2.48 ) | 1.49 ( 2.41 ) | 1.49 ( 0.97 ) | 0.57 ( -0.15 ) |
| Renal failure* | 15 | 2.6 ( 1.56 - 4.33 ) | 2.59 ( 14.57 ) | 2.58 ( 1.68 ) | 1.37 ( 0.64 ) |
| Weight decreased* | 15 | 1.73 ( 1.04 - 2.87 ) | 1.72 ( 4.52 ) | 1.72 ( 1.12 ) | 0.78 ( 0.05 ) |
| Colitis* | 15 | 20.54 ( 12.17 - 34.64 ) | 20.39 ( 261.1 ) | 19.3 ( 12.46 ) | 4.27 ( 3.52 ) |
| Nausea | 14 | 0.88 ( 0.52 - 1.49 ) | 0.88 ( 0.22 ) | 0.88 ( 0.57 ) | -0.18 ( -0.93 ) |
| Fall | 14 | 0.35 ( 0.21 - 0.59 ) | 0.36 ( 16.61 ) | 0.36 ( 0.23 ) | -1.49 ( -2.24 ) |
| Muscular weakness* | 14 | 3.28 ( 1.93 - 5.56 ) | 3.26 ( 21.83 ) | 3.24 ( 2.08 ) | 1.7 ( 0.95 ) |
| Malaise | 14 | 0.89 ( 0.53 - 1.51 ) | 0.89 ( 0.19 ) | 0.89 ( 0.57 ) | -0.17 ( -0.91 ) |
| Anaemia | 13 | 0.97 ( 0.56 - 1.67 ) | 0.97 ( 0.01 ) | 0.97 ( 0.61 ) | -0.04 ( -0.82 ) |
| Pneumonia aspiration* | 13 | 3.92 ( 2.26 - 6.78 ) | 3.9 ( 27.75 ) | 3.87 ( 2.44 ) | 1.95 ( 1.17 ) |
| Adverse event* | 13 | 5.22 ( 3.01 - 9.04 ) | 5.19 ( 43.37 ) | 5.13 ( 3.24 ) | 2.36 ( 1.58 ) |
| Urinary tract infection | 12 | 1.16 ( 0.65 - 2.04 ) | 1.15 ( 0.25 ) | 1.15 ( 0.72 ) | 0.21 ( -0.6 ) |
| Pemphigoid* | 12 | 7.83 ( 4.41 - 13.89 ) | 7.78 ( 69.43 ) | 7.63 ( 4.72 ) | 2.93 ( 2.12 ) |
| Pleural effusion* | 11 | 3.39 ( 1.87 - 6.15 ) | 3.38 ( 18.27 ) | 3.36 ( 2.04 ) | 1.75 ( 0.91 ) |
| Hepatic function abnormal* | 11 | 7.97 ( 4.38 - 14.52 ) | 7.93 ( 65.17 ) | 7.77 ( 4.71 ) | 2.96 ( 2.11 ) |
| Myositis* | 11 | 50.51 ( 26.78 - 95.26 ) | 50.23 ( 462.94 ) | 43.93 ( 25.84 ) | 5.46 ( 4.57 ) |
| Prescribed underdose* | 10 | 6.03 ( 3.22 - 11.29 ) | 6.01 ( 41.07 ) | 5.92 ( 3.51 ) | 2.57 ( 1.69 ) |
| Hypertension | 10 | 1.44 ( 0.77 - 2.69 ) | 1.44 ( 1.34 ) | 1.44 ( 0.85 ) | 0.52 ( -0.35 ) |
| General physical health deterioration | 10 | 1.32 ( 0.71 - 2.45 ) | 1.31 ( 0.75 ) | 1.31 ( 0.78 ) | 0.39 ( -0.48 ) |
| Pulmonary embolism* | 9 | 3.63 ( 1.88 - 7.02 ) | 3.62 ( 16.93 ) | 3.59 ( 2.07 ) | 1.85 ( 0.93 ) |
| Infusion related reaction* | 9 | 9.98 ( 5.14 - 19.4 ) | 9.94 ( 70.39 ) | 9.69 ( 5.56 ) | 3.28 ( 2.35 ) |
| Gait disturbance | 9 | 1.2 ( 0.62 - 2.31 ) | 1.2 ( 0.3 ) | 1.2 ( 0.69 ) | 0.26 ( -0.65 ) |
| Dehydration | 9 | 1.02 ( 0.53 - 1.97 ) | 1.02 ( 0 ) | 1.02 ( 0.59 ) | 0.03 ( -0.89 ) |
| Pain | 9 | 0.9 ( 0.47 - 1.74 ) | 0.9 ( 0.1 ) | 0.9 ( 0.52 ) | -0.15 ( -1.06 ) |
| Dysphagia | 9 | 1.5 ( 0.78 - 2.89 ) | 1.49 ( 1.47 ) | 1.49 ( 0.86 ) | 0.58 ( -0.34 ) |
| Diabetic ketoacidosis* | 9 | 34.02 ( 17.13 - 67.59 ) | 33.87 ( 261.32 ) | 30.91 ( 17.41 ) | 4.95 ( 3.99 ) |
| Drug ineffective | 9 | 0.34 ( 0.18 - 0.65 ) | 0.34 ( 11.55 ) | 0.34 ( 0.2 ) | -1.55 ( -2.46 ) |
| Myasthenia gravis* | 9 | 50.76 ( 25.17 - 102.35 ) | 50.53 ( 380.82 ) | 44.16 ( 24.56 ) | 5.46 ( 4.49 ) |
| Product use in unapproved Indication | 8 | 0.9 ( 0.45 - 1.8 ) | 0.9 ( 0.09 ) | 0.9 ( 0.5 ) | -0.15 ( -1.12 ) |
| adrenal insufficiency* | 8 | 30.91 ( 14.97 - 63.8 ) | 30.79 ( 211.56 ) | 28.33 ( 15.45 ) | 4.82 ( 3.82 ) |
| Taste disorder* | 8 | 13.03 ( 6.43 - 26.44 ) | 12.99 ( 85.29 ) | 12.55 ( 6.94 ) | 3.65 ( 2.67 ) |
| Covid-19 | 8 | 1.27 ( 0.63 - 2.55 ) | 1.27 ( 0.46 ) | 1.27 ( 0.71 ) | 0.35 ( -0.62 ) |

Abbreviation: Asterisks (*) indicate statistically significant signals in algorithm; ROR, reporting odds ratio; PRR, proportional reporting ratio; EBGM, empirical Bayesian geometric mean; EBGM05, the lower limit of the 95% CI of EBGM; IC, information component; IC025, the lower limit of the 95% CI of the IC; CI, confidence interval; PT, preferred term.

Supplementary Table 10:

Top 50 most frequent adverse events for Nivolumab at the PT level in patients reported by medical personnels from FAERS data

| PT | Case numbers | ROR(95%CI) | PRR(χ^2^) | EBGM(EBGM05) | IC(IC025) |
| --- | --- | --- | --- | --- | --- |
| Malignant neoplasm progression* | 5937 | 22.46 ( 21.85 - 23.1 ) | 21.44 ( 101705.48 ) | 18.92 ( 18.49 ) | 4.24 ( 4.2 ) |
| Death* | 5375 | 3.17 ( 3.08 - 3.26 ) | 3.07 ( 7476.2 ) | 3.03 ( 2.96 ) | 1.6 ( 1.56 ) |
| Off label use | 2256 | 0.9 ( 0.86 - 0.94 ) | 0.9 ( 23.88 ) | 0.9 ( 0.87 ) | -0.15 ( -0.21 ) |
| Intentional product use issue* | 2055 | 7.54 ( 7.21 - 7.88 ) | 7.43 ( 10925 ) | 7.13 ( 6.87 ) | 2.83 ( 2.77 ) |
| Diarrhoea* | 1988 | 1.43 ( 1.37 - 1.49 ) | 1.42 ( 250.19 ) | 1.42 ( 1.37 ) | 0.5 ( 0.44 ) |
| Pyrexia* | 1719 | 2.07 ( 1.97 - 2.17 ) | 2.05 ( 922.08 ) | 2.04 ( 1.96 ) | 1.03 ( 0.96 ) |
| Dyspnoea* | 1344 | 1.18 ( 1.12 - 1.25 ) | 1.18 ( 37 ) | 1.18 ( 1.13 ) | 0.24 ( 0.16 ) |
| Pneumonia* | 1252 | 1.62 ( 1.53 - 1.72 ) | 1.62 ( 293.24 ) | 1.61 ( 1.54 ) | 0.69 ( 0.6 ) |
| Fatigue | 1244 | 0.87 ( 0.82 - 0.92 ) | 0.87 ( 24.21 ) | 0.87 ( 0.83 ) | -0.2 ( -0.28 ) |
| Colitis* | 1183 | 12.22 ( 11.52 - 12.97 ) | 12.12 ( 11189.44 ) | 11.3 ( 10.75 ) | 3.5 ( 3.41 ) |
| Rash* | 1090 | 1.12 ( 1.05 - 1.19 ) | 1.12 ( 13 ) | 1.11 ( 1.06 ) | 0.16 ( 0.07 ) |
| Pneumonitis* | 1062 | 12.71 ( 11.94 - 13.54 ) | 12.61 ( 10497.47 ) | 11.73 ( 11.13 ) | 3.55 ( 3.46 ) |
| Nausea | 1021 | 0.67 ( 0.63 - 0.72 ) | 0.68 ( 158.44 ) | 0.68 ( 0.64 ) | -0.56 ( -0.65 ) |
| Acute kidney injury* | 1012 | 1.69 ( 1.59 - 1.8 ) | 1.69 ( 280.25 ) | 1.68 ( 1.59 ) | 0.75 ( 0.66 ) |
| Decreased appetite* | 994 | 2.15 ( 2.02 - 2.29 ) | 2.14 ( 597.11 ) | 2.12 ( 2.01 ) | 1.09 ( 0.99 ) |
| Hypothyroidism* | 975 | 13.59 ( 12.72 - 14.51 ) | 13.49 ( 10364.77 ) | 12.47 ( 11.81 ) | 3.64 ( 3.54 ) |
| Anaemia* | 866 | 1.65 ( 1.54 - 1.77 ) | 1.65 ( 218.98 ) | 1.64 ( 1.55 ) | 0.71 ( 0.62 ) |
| Vomiting | 845 | 0.9 ( 0.84 - 0.96 ) | 0.9 ( 9.79 ) | 0.9 ( 0.85 ) | -0.15 ( -0.25 ) |
| General physical health deterioration* | 834 | 2.91 ( 2.71 - 3.11 ) | 2.9 ( 1017.7 ) | 2.86 ( 2.7 ) | 1.52 ( 1.41 ) |
| Asthenia* | 822 | 1.22 ( 1.14 - 1.31 ) | 1.22 ( 33.03 ) | 1.22 ( 1.15 ) | 0.29 ( 0.19 ) |
| Interstitial lung disease* | 794 | 4.94 ( 4.6 - 5.3 ) | 4.91 ( 2399.6 ) | 4.79 ( 4.51 ) | 2.26 ( 2.16 ) |
| Sepsis* | 694 | 2.24 ( 2.08 - 2.42 ) | 2.24 ( 468.24 ) | 2.22 ( 2.08 ) | 1.15 ( 1.04 ) |
| Immune-mediated enterocolitis* | 665 | 70.26 ( 64.09 - 77.02 ) | 69.89 ( 30980.25 ) | 48.26 ( 44.68 ) | 5.59 ( 5.46 ) |
| Product use in unapproved indication | 644 | 1 ( 0.93 - 1.08 ) | 1 ( 0 ) | 1 ( 0.94 ) | 0 ( -0.11 ) |
| Arthralgia | 626 | 0.75 ( 0.69 - 0.81 ) | 0.75 ( 52.81 ) | 0.75 ( 0.7 ) | -0.41 ( -0.53 ) |
| Weight decreased* | 620 | 1.27 ( 1.17 - 1.38 ) | 1.27 ( 35.3 ) | 1.27 ( 1.19 ) | 0.34 ( 0.23 ) |
| Adrenal insufficiency* | 614 | 16.66 ( 15.33 - 18.11 ) | 16.58 ( 8112.64 ) | 15.06 ( 14.04 ) | 3.91 ( 3.79 ) |
| Malaise | 610 | 0.81 ( 0.74 - 0.87 ) | 0.81 ( 28.14 ) | 0.81 ( 0.76 ) | -0.31 ( -0.42 ) |
| Myocarditis* | 604 | 16.41 ( 15.09 - 17.85 ) | 16.34 ( 7857.87 ) | 14.85 ( 13.84 ) | 3.89 ( 3.77 ) |
| Pruritus | 604 | 0.8 ( 0.74 - 0.87 ) | 0.8 ( 29.07 ) | 0.8 ( 0.75 ) | -0.31 ( -0.43 ) |
| Back pain* | 594 | 1.44 ( 1.33 - 1.57 ) | 1.44 ( 79.92 ) | 1.44 ( 1.34 ) | 0.52 ( 0.4 ) |
| Pleural effusion* | 585 | 3.74 ( 3.44 - 4.06 ) | 3.72 ( 1138.97 ) | 3.66 ( 3.42 ) | 1.87 ( 1.75 ) |
| Hepatic function abnormal* | 528 | 4.76 ( 4.36 - 5.19 ) | 4.74 ( 1514 ) | 4.63 ( 4.31 ) | 2.21 ( 2.08 ) |
| Headache | 526 | 0.47 ( 0.44 - 0.52 ) | 0.48 ( 303.48 ) | 0.48 ( 0.45 ) | -1.06 ( -1.19 ) |
| Adverse event* | 515 | 3.46 ( 3.17 - 3.78 ) | 3.45 ( 878.28 ) | 3.4 ( 3.16 ) | 1.76 ( 1.64 ) |
| Dehydration* | 506 | 2 ( 1.83 - 2.18 ) | 1.99 ( 248.35 ) | 1.98 ( 1.84 ) | 0.99 ( 0.86 ) |
| Respiratory failure* | 503 | 2.43 ( 2.23 - 2.66 ) | 2.43 ( 416.33 ) | 2.41 ( 2.23 ) | 1.27 ( 1.14 ) |
| Pemphigoid* | 496 | 19.76 ( 18 - 21.7 ) | 19.69 ( 7795.28 ) | 17.55 ( 16.23 ) | 4.13 ( 4 ) |
| Thrombocytopenia* | 490 | 1.3 ( 1.19 - 1.42 ) | 1.29 ( 32.71 ) | 1.29 ( 1.2 ) | 0.37 ( 0.24 ) |
| Pain | 482 | 0.53 ( 0.49 - 0.58 ) | 0.54 ( 195.51 ) | 0.54 ( 0.5 ) | -0.9 ( -1.03 ) |
| Hypotension | 475 | 0.9 ( 0.82 - 0.98 ) | 0.9 ( 5.76 ) | 0.9 ( 0.83 ) | -0.16 ( -0.29 ) |
| Cough | 460 | 0.88 ( 0.8 - 0.96 ) | 0.88 ( 7.67 ) | 0.88 ( 0.81 ) | -0.19 ( -0.32 ) |
| Hyperthyroidism* | 459 | 12.9 ( 11.72 - 14.19 ) | 12.85 ( 4629.27 ) | 11.93 ( 11.02 ) | 3.58 ( 3.44 ) |
| Renal failure* | 459 | 1.98 ( 1.8 - 2.17 ) | 1.97 ( 217.4 ) | 1.96 ( 1.81 ) | 0.97 ( 0.83 ) |
| Abdominal pain | 455 | 0.9 ( 0.82 - 0.99 ) | 0.9 ( 4.59 ) | 0.9 ( 0.84 ) | -0.14 ( -0.28 ) |
| Liver disorder* | 450 | 4.98 ( 4.53 - 5.47 ) | 4.97 ( 1382.05 ) | 4.84 ( 4.48 ) | 2.28 ( 2.14 ) |
| Hepatitis* | 442 | 6.47 ( 5.88 - 7.11 ) | 6.45 ( 1952.83 ) | 6.23 ( 5.75 ) | 2.64 ( 2.5 ) |
| Hyponatraemia* | 433 | 2.36 ( 2.15 - 2.6 ) | 2.36 ( 334.47 ) | 2.34 ( 2.16 ) | 1.23 ( 1.09 ) |
| Febrile neutropenia* | 423 | 1.67 ( 1.52 - 1.84 ) | 1.67 ( 112.86 ) | 1.66 ( 1.53 ) | 0.73 ( 0.59 ) |
| Infusion related reaction* | 416 | 1.91 ( 1.73 - 2.1 ) | 1.9 ( 176.85 ) | 1.89 ( 1.75 ) | 0.92 ( 0.78 ) |

Abbreviation: Asterisks (*) indicate statistically significant signals in algorithm; ROR, reporting odds ratio; PRR, proportional reporting ratio; EBGM, empirical Bayesian geometric mean; EBGM05, the lower limit of the 95% CI of EBGM; IC, information component; IC025, the lower limit of the 95% CI of the IC; CI, confidence interval; PT, preferred term.

Supplementary Table 11:

Top 50 most frequent adverse events for Nivolumab at the PT level in patients reported by non-medical personnels from FAERS data

| PT | Case numbers | ROR(95%CI) | PRR(χ^2^) | EBGM(EBGM05) | IC(IC025) |
| --- | --- | --- | --- | --- | --- |
| Death* | 4617 | 7.42 ( 7.19 - 7.65 ) | 6.78 ( 22726.48 ) | 6.69 ( 6.52 ) | 2.74 ( 2.7 ) |
| Malignant neoplasm progression* | 1594 | 32.96 ( 31.3 - 34.71 ) | 31.87 ( 44321.54 ) | 29.67 ( 28.41 ) | 4.89 ( 4.82 ) |
| Fatigue* | 1287 | 1.81 ( 1.71 - 1.91 ) | 1.79 ( 451.16 ) | 1.78 ( 1.7 ) | 0.83 ( 0.75 ) |
| Off label use* | 1276 | 1.99 ( 1.89 - 2.11 ) | 1.97 ( 612.49 ) | 1.96 ( 1.87 ) | 0.97 ( 0.89 ) |
| Intentional product use issue* | 1038 | 15.64 ( 14.69 - 16.66 ) | 15.32 ( 13418.88 ) | 14.81 ( 14.05 ) | 3.89 ( 3.8 ) |
| Diarrhoea* | 928 | 1.84 ( 1.73 - 1.97 ) | 1.83 ( 348.96 ) | 1.82 ( 1.73 ) | 0.87 ( 0.77 ) |
| Decreased appetite* | 656 | 3.47 ( 3.21 - 3.75 ) | 3.43 ( 1126.24 ) | 3.41 ( 3.2 ) | 1.77 ( 1.66 ) |
| Nausea | 644 | 1.07 ( 0.99 - 1.16 ) | 1.07 ( 3.16 ) | 1.07 ( 1 ) | 0.1 ( -0.01 ) |
| Rash* | 621 | 2.05 ( 1.89 - 2.22 ) | 2.03 ( 326.48 ) | 2.03 ( 1.9 ) | 1.02 ( 0.9 ) |
| Asthenia* | 580 | 1.91 ( 1.76 - 2.07 ) | 1.9 ( 245.89 ) | 1.89 ( 1.77 ) | 0.92 ( 0.8 ) |
| Dyspnoea* | 550 | 1.31 ( 1.21 - 1.43 ) | 1.31 ( 40.11 ) | 1.31 ( 1.22 ) | 0.39 ( 0.26 ) |
| Weight decreased* | 548 | 2.29 ( 2.1 - 2.49 ) | 2.27 ( 390.46 ) | 2.27 ( 2.11 ) | 1.18 ( 1.06 ) |
| Adverse event* | 523 | 6.08 ( 5.57 - 6.63 ) | 6.02 ( 2162.19 ) | 5.95 ( 5.53 ) | 2.57 ( 2.44 ) |
| Pruritus* | 495 | 1.78 ( 1.63 - 1.94 ) | 1.77 ( 165.75 ) | 1.77 ( 1.64 ) | 0.82 ( 0.69 ) |
| Pyrexia* | 490 | 2.59 ( 2.37 - 2.83 ) | 2.57 ( 468.85 ) | 2.56 ( 2.38 ) | 1.36 ( 1.22 ) |
| Pain | 391 | 0.58 ( 0.53 - 0.65 ) | 0.59 ( 114.8 ) | 0.59 ( 0.54 ) | -0.77 ( -0.91 ) |
| Product use in unapproved indication* | 390 | 1.75 ( 1.59 - 1.94 ) | 1.75 ( 124.47 ) | 1.74 ( 1.6 ) | 0.8 ( 0.65 ) |
| Arthralgia* | 386 | 1.11 ( 1.01 - 1.23 ) | 1.11 ( 4.36 ) | 1.11 ( 1.02 ) | 0.15 ( 0.01 ) |
| Constipation* | 385 | 2.07 ( 1.87 - 2.28 ) | 2.06 ( 208.81 ) | 2.05 ( 1.89 ) | 1.04 ( 0.89 ) |
| Cough* | 369 | 1.49 ( 1.34 - 1.65 ) | 1.48 ( 58.15 ) | 1.48 ( 1.36 ) | 0.57 ( 0.42 ) |
| Pneumonia* | 368 | 1.81 ( 1.64 - 2.01 ) | 1.81 ( 132.76 ) | 1.8 ( 1.66 ) | 0.85 ( 0.7 ) |
| Vomiting* | 366 | 1.17 ( 1.05 - 1.29 ) | 1.17 ( 8.72 ) | 1.17 ( 1.07 ) | 0.22 ( 0.07 ) |
| Headache | 346 | 0.63 ( 0.56 - 0.7 ) | 0.63 ( 76.15 ) | 0.63 ( 0.58 ) | -0.67 ( -0.82 ) |
| Drug ineffective | 338 | 0.25 ( 0.22 - 0.28 ) | 0.25 ( 762.23 ) | 0.25 ( 0.23 ) | -1.97 ( -2.13 ) |
| Malaise | 292 | 0.69 ( 0.62 - 0.78 ) | 0.69 ( 39.76 ) | 0.69 ( 0.63 ) | -0.53 ( -0.7 ) |
| Blood pressure increased* | 255 | 2.01 ( 1.77 - 2.27 ) | 2 ( 127.2 ) | 2 ( 1.8 ) | 1 ( 0.82 ) |
| Product use issue | 238 | 1.06 ( 0.93 - 1.2 ) | 1.06 ( 0.81 ) | 1.06 ( 0.95 ) | 0.08 ( -0.1 ) |
| General physical health deterioration* | 237 | 4 ( 3.52 - 4.54 ) | 3.98 ( 524.64 ) | 3.95 ( 3.55 ) | 1.98 ( 1.79 ) |
| Pneumonitis* | 236 | 27.04 ( 23.7 - 30.85 ) | 26.91 ( 5530.04 ) | 25.33 ( 22.68 ) | 4.66 ( 4.47 ) |
| Colitis* | 233 | 12.66 ( 11.11 - 14.42 ) | 12.6 ( 2415.72 ) | 12.26 ( 10.99 ) | 3.62 ( 3.42 ) |
| Pain in extremity | 231 | 0.86 ( 0.76 - 0.98 ) | 0.86 ( 5.02 ) | 0.86 ( 0.77 ) | -0.21 ( -0.4 ) |
| Back pain | 223 | 1.11 ( 0.97 - 1.27 ) | 1.11 ( 2.49 ) | 1.11 ( 1 ) | 0.15 ( -0.04 ) |
| Dizziness | 214 | 0.5 ( 0.43 - 0.57 ) | 0.5 ( 108.3 ) | 0.5 ( 0.45 ) | -1 ( -1.2 ) |
| Autoimmune disorder* | 198 | 29.04 ( 25.13 - 33.55 ) | 28.92 ( 4990.28 ) | 27.1 ( 24.02 ) | 4.76 ( 4.55 ) |
| Myalgia* | 190 | 1.53 ( 1.33 - 1.77 ) | 1.53 ( 35.13 ) | 1.53 ( 1.36 ) | 0.61 ( 0.4 ) |
| Gait disturbance | 182 | 0.96 ( 0.83 - 1.11 ) | 0.96 ( 0.32 ) | 0.96 ( 0.85 ) | -0.06 ( -0.27 ) |
| Fall | 181 | 0.67 ( 0.58 - 0.77 ) | 0.67 ( 30.19 ) | 0.67 ( 0.59 ) | -0.58 ( -0.8 ) |
| Taste disorder* | 177 | 8.17 ( 7.04 - 9.49 ) | 8.15 ( 1088.86 ) | 8.01 ( 7.07 ) | 3 ( 2.78 ) |
| Anaemia* | 176 | 2.29 ( 1.98 - 2.66 ) | 2.29 ( 126.87 ) | 2.28 ( 2.01 ) | 1.19 ( 0.97 ) |
| Hypothyroidism* | 172 | 10.99 ( 9.44 - 12.78 ) | 10.95 ( 1515.52 ) | 10.69 ( 9.42 ) | 3.42 ( 3.2 ) |
| Peripheral swelling | 164 | 0.96 ( 0.82 - 1.12 ) | 0.96 ( 0.31 ) | 0.96 ( 0.84 ) | -0.06 ( -0.29 ) |
| Dry skin | 163 | 0.94 ( 0.81 - 1.1 ) | 0.94 ( 0.53 ) | 0.94 ( 0.83 ) | -0.08 ( -0.31 ) |
| Somnolence | 159 | 0.97 ( 0.83 - 1.13 ) | 0.97 ( 0.15 ) | 0.97 ( 0.85 ) | -0.04 ( -0.27 ) |
| Abdominal pain upper | 158 | 0.85 ( 0.72 - 0.99 ) | 0.85 ( 4.38 ) | 0.85 ( 0.74 ) | -0.24 ( -0.47 ) |
| Hospitalisation* | 158 | 1.27 ( 1.09 - 1.49 ) | 1.27 ( 9.19 ) | 1.27 ( 1.12 ) | 0.35 ( 0.12 ) |
| Stomatitis* | 151 | 4.05 ( 3.45 - 4.76 ) | 4.04 ( 342.8 ) | 4.01 ( 3.51 ) | 2 ( 1.77 ) |
| Blister* | 148 | 3.41 ( 2.9 - 4.01 ) | 3.4 ( 249.34 ) | 3.38 ( 2.95 ) | 1.76 ( 1.52 ) |
| Dehydration* | 146 | 1.72 ( 1.46 - 2.02 ) | 1.72 ( 43.6 ) | 1.71 ( 1.5 ) | 0.78 ( 0.54 ) |
| Abdominal pain | 144 | 0.99 ( 0.84 - 1.17 ) | 0.99 ( 0.01 ) | 0.99 ( 0.86 ) | -0.01 ( -0.25 ) |
| Thyroid disorder* | 144 | 9.72 ( 8.24 - 11.47 ) | 9.69 ( 1097.03 ) | 9.49 ( 8.26 ) | 3.25 ( 3 ) |

Abbreviation: Asterisks (*) indicate statistically significant signals in algorithm; ROR, reporting odds ratio; PRR, proportional reporting ratio; EBGM, empirical Bayesian geometric mean; EBGM05, the lower limit of the 95% CI of EBGM; IC, information component; IC025, the lower limit of the 95% CI of the IC; CI, confidence interval; PT, preferred term.

Supplementary Table 12:

Top 50 most frequent adverse events for Nivolumab excluding common medication co-usage at the PT level from FAERS data

| PT | Case numbers | ROR(95%CI) | PRR(χ^2^) | EBGM(EBGM05) | IC(IC025) |
| --- | --- | --- | --- | --- | --- |
| Death* | 7878 | 4.52 ( 4.41 - 4.62 ) | 4.3 ( 19947.25 ) | 4.25 ( 4.17 ) | 2.09 ( 2.05 ) |
| Malignant neoplasm progression* | 6077 | 29.8 ( 29.01 - 30.61 ) | 28.42 ( 147584.57 ) | 26.13 ( 25.54 ) | 4.71 ( 4.67 ) |
| Off label use* | 2459 | 1.16 ( 1.11 - 1.21 ) | 1.16 ( 52.08 ) | 1.15 ( 1.12 ) | 0.21 ( 0.15 ) |
| Intentional product use issue* | 2036 | 8.8 ( 8.42 - 9.2 ) | 8.67 ( 13469.26 ) | 8.46 ( 8.16 ) | 3.08 ( 3.02 ) |
| Diarrhoea* | 1943 | 1.4 ( 1.34 - 1.46 ) | 1.39 ( 214.98 ) | 1.39 ( 1.34 ) | 0.47 ( 0.41 ) |
| Fatigue* | 1814 | 1.06 ( 1.02 - 1.12 ) | 1.06 ( 7.07 ) | 1.06 ( 1.02 ) | 0.09 ( 0.02 ) |
| Pyrexia* | 1464 | 2.16 ( 2.05 - 2.28 ) | 2.15 ( 897.41 ) | 2.14 ( 2.05 ) | 1.1 ( 1.02 ) |
| Dyspnoea* | 1462 | 1.27 ( 1.21 - 1.34 ) | 1.27 ( 83.13 ) | 1.27 ( 1.21 ) | 0.34 ( 0.27 ) |
| Pneumonia* | 1282 | 1.93 ( 1.82 - 2.04 ) | 1.92 ( 561.95 ) | 1.91 ( 1.83 ) | 0.93 ( 0.85 ) |
| Decreased appetite* | 1225 | 2.49 ( 2.35 - 2.63 ) | 2.47 ( 1071.24 ) | 2.46 ( 2.35 ) | 1.3 ( 1.22 ) |
| Nausea | 1184 | 0.74 ( 0.7 - 0.79 ) | 0.75 ( 104.35 ) | 0.75 ( 0.71 ) | -0.42 ( -0.51 ) |
| Rash* | 1142 | 1.26 ( 1.19 - 1.34 ) | 1.26 ( 61.96 ) | 1.26 ( 1.2 ) | 0.33 ( 0.25 ) |
| Asthenia* | 1095 | 1.45 ( 1.37 - 1.54 ) | 1.45 ( 152.17 ) | 1.45 ( 1.38 ) | 0.53 ( 0.44 ) |
| Pneumonitis* | 918 | 16.7 ( 15.63 - 17.85 ) | 16.59 ( 12772.99 ) | 15.8 ( 14.94 ) | 3.98 ( 3.88 ) |
| Weight decreased* | 872 | 1.51 ( 1.41 - 1.62 ) | 1.51 ( 149.69 ) | 1.51 ( 1.42 ) | 0.59 ( 0.49 ) |
| Hypothyroidism* | 866 | 14.85 ( 13.87 - 15.9 ) | 14.76 ( 10609.66 ) | 14.14 ( 13.35 ) | 3.82 ( 3.72 ) |
| Vomiting | 831 | 0.92 ( 0.86 - 0.98 ) | 0.92 ( 6.29 ) | 0.92 ( 0.87 ) | -0.12 ( -0.23 ) |
| General physical health deterioration* | 821 | 3.69 ( 3.44 - 3.95 ) | 3.67 ( 1580.48 ) | 3.64 ( 3.44 ) | 1.86 ( 1.76 ) |
| Anaemia* | 804 | 2.2 ( 2.05 - 2.35 ) | 2.19 ( 517.09 ) | 2.18 ( 2.06 ) | 1.12 ( 1.02 ) |
| Arthralgia | 790 | 0.89 ( 0.83 - 0.95 ) | 0.89 ( 11.2 ) | 0.89 ( 0.84 ) | -0.17 ( -0.27 ) |
| Adverse event* | 784 | 4.09 ( 3.81 - 4.39 ) | 4.07 ( 1798.34 ) | 4.04 ( 3.8 ) | 2.01 ( 1.91 ) |
| Product use in unapproved indication* | 782 | 1.26 ( 1.18 - 1.36 ) | 1.26 ( 42.74 ) | 1.26 ( 1.19 ) | 0.34 ( 0.23 ) |
| Pruritus | 778 | 1.03 ( 0.96 - 1.1 ) | 1.03 ( 0.56 ) | 1.03 ( 0.97 ) | 0.04 ( -0.07 ) |
| Interstitial lung disease* | 769 | 8.15 ( 7.59 - 8.76 ) | 8.11 ( 4675.15 ) | 7.93 ( 7.47 ) | 2.99 ( 2.88 ) |
| Malaise | 703 | 0.73 ( 0.68 - 0.79 ) | 0.74 ( 67.52 ) | 0.74 ( 0.69 ) | -0.44 ( -0.55 ) |
| Acute kidney injury* | 691 | 1.65 ( 1.53 - 1.78 ) | 1.65 ( 175.14 ) | 1.64 ( 1.54 ) | 0.72 ( 0.61 ) |
| Colitis* | 667 | 8.74 ( 8.09 - 9.44 ) | 8.7 ( 4426.34 ) | 8.49 ( 7.96 ) | 3.09 ( 2.97 ) |
| Pain | 667 | 0.49 ( 0.45 - 0.53 ) | 0.49 ( 354.64 ) | 0.49 ( 0.46 ) | -1.02 ( -1.14 ) |
| Back pain* | 623 | 1.3 ( 1.2 - 1.4 ) | 1.29 ( 41.49 ) | 1.29 ( 1.21 ) | 0.37 ( 0.25 ) |
| Cough | 617 | 1.02 ( 0.94 - 1.1 ) | 1.02 ( 0.25 ) | 1.02 ( 0.95 ) | 0.03 ( -0.09 ) |
| Constipation* | 616 | 1.37 ( 1.26 - 1.48 ) | 1.37 ( 60.37 ) | 1.36 ( 1.28 ) | 0.45 ( 0.33 ) |
| Pleural effusion* | 554 | 4.91 ( 4.51 - 5.34 ) | 4.89 ( 1689.44 ) | 4.83 ( 4.5 ) | 2.27 ( 2.15 ) |
| Sepsis* | 548 | 2.51 ( 2.31 - 2.73 ) | 2.5 ( 490.69 ) | 2.49 ( 2.32 ) | 1.32 ( 1.19 ) |
| Headache | 533 | 0.4 ( 0.37 - 0.44 ) | 0.41 ( 467.61 ) | 0.41 ( 0.38 ) | -1.3 ( -1.42 ) |
| Thrombocytopenia | *514 | 2.39 ( 2.19 - 2.61 ) | 2.39 ( 411.78 ) | 2.38 ( 2.21 ) | 1.25 ( 1.12 ) |
| Product use issue | 487 | 0.95 ( 0.87 - 1.04 ) | 0.95 ( 1.07 ) | 0.95 ( 0.89 ) | -0.07 ( -0.2 ) |
| Respiratory failure* | 477 | 3.62 ( 3.31 - 3.97 ) | 3.61 ( 892.35 ) | 3.58 ( 3.32 ) | 1.84 ( 1.71 ) |
| Fall | 464 | 0.69 ( 0.63 - 0.75 ) | 0.69 ( 66.22 ) | 0.69 ( 0.64 ) | -0.54 ( -0.67 ) |
| Pemphigoid* | 459 | 31.71 ( 28.8 - 34.91 ) | 31.6 ( 12349.36 ) | 28.78 ( 26.56 ) | 4.85 ( 4.71 ) |
| Drug ineffective | 450 | 0.15 ( 0.13 - 0.16 ) | 0.15 ( 2225.81 ) | 0.15 ( 0.14 ) | -2.74 ( -2.87 ) |
| Abdominal pain | 422 | 0.94 ( 0.85 - 1.03 ) | 0.94 ( 1.86 ) | 0.94 ( 0.86 ) | -0.1 ( -0.24 ) |
| Lung disorder* | 420 | 4.27 ( 3.88 - 4.7 ) | 4.26 ( 1034.32 ) | 4.22 ( 3.89 ) | 2.08 ( 1.93 ) |
| Prescribed underdose* | 418 | 8.22 ( 7.46 - 9.06 ) | 8.2 ( 2574.69 ) | 8.01 ( 7.39 ) | 3 ( 2.86 ) |
| Myocarditis* | 418 | 18.94 ( 17.16 - 20.91 ) | 18.88 ( 6676.14 ) | 17.86 ( 16.44 ) | 4.16 ( 4.01 ) |
| Adrenal insufficiency* | 415 | 17.33 ( 15.69 - 19.13 ) | 17.27 ( 6029.09 ) | 16.42 ( 15.11 ) | 4.04 ( 3.89 ) |
| Hepatic function abnormal* | 415 | 6.05 ( 5.49 - 6.67 ) | 6.04 ( 1711.65 ) | 5.94 ( 5.48 ) | 2.57 ( 2.43 ) |
| Dehydration* | 413 | 1.68 ( 1.53 - 1.85 ) | 1.68 ( 112.79 ) | 1.67 ( 1.54 ) | 0.74 ( 0.6 ) |
| Dizziness | 401 | 0.4 ( 0.36 - 0.44 ) | 0.4 ( 359.99 ) | 0.4 ( 0.37 ) | -1.31 ( -1.46 ) |
| Infusion related reaction* | 396 | 2.89 ( 2.62 - 3.19 ) | 2.88 ( 483.75 ) | 2.87 ( 2.64 ) | 1.52 ( 1.37 ) |
| Renal failure* | 396 | 1.48 ( 1.34 - 1.64 ) | 1.48 ( 62.13 ) | 1.48 ( 1.36 ) | 0.57 ( 0.42 ) |

Abbreviation: Asterisks (*) indicate statistically significant signals in algorithm; ROR, reporting odds ratio; PRR, proportional reporting ratio; EBGM, empirical Bayesian geometric mean; EBGM05, the lower limit of the 95% CI of EBGM; IC, information component; IC025, the lower limit of the 95% CI of the IC; CI, confidence interval; PT, preferred term.
